# CD105^+^CD90^+^CD13^+^ identifies a clonogenic subset of adventitial lung fibroblasts

**DOI:** 10.1038/s41598-021-03963-9

**Published:** 2021-12-24

**Authors:** Måns Kadefors, Sara Rolandsson Enes, Emma Åhrman, Barbora Michaliková, Anna Löfdahl, Göran Dellgren, Stefan Scheding, Gunilla Westergren-Thorsson

**Affiliations:** 1 Department of Experimental Medical Science, Lund University, Lund, Sweden; 2 Division of Infection Medicine, Department of Clinical Sciences Lund, Lund University, Lund, Sweden; 3 Department of Cardiothoracic Surgery and Transplant Institute, Sahlgrenska University Hospital, Göteborg, Sweden; 4 Division of Molecular Hematology, Lund Stem Cell Center, Lund University, Lund, Sweden; 5 Department of Hematology, Skåne University Hospital Lund, Lund, Sweden

**Keywords:** Cell biology, Molecular biology, Preclinical research

## Abstract

Mesenchymal cells are important components of specified niches in the lung, and can mediate a wide range of processes including tissue regeneration and repair. Dysregulation of these processes can lead to improper remodeling of tissue as observed in several lung diseases. The mesenchymal cells responsible remain poorly described, partially due to the heterogenic nature of the mesenchymal compartment and the absence of appropriate markers. Here, we describe that CD105^+^CD90^+^ mesenchymal cells can be divided into two populations based on their expression of CD13/aminopeptidase N (CD105^+^CD90^+^CD13^−^ and CD105^+^CD90^+^CD13^+^). By prospective isolation using FACS, we show that both these populations give rise to clonogenic fibroblast-like cells, but with an increased clonogenic and proliferative capacity of CD105^+^CD90^+^CD13^+^ cells. Transcriptomic and spatial analysis pinpoints an adventitial fibroblast subset as the origin of CD105^+^CD90^+^CD13^+^ clonogenic mesenchymal cells in human lung.

## Introduction

The lung mesenchyme contains a heterogeneous group of mesenchymal cells that can serve as important mediators for tissue repair and regeneration and provide an important niche for endothelial and epithelial cell function. As residents of tissue stroma mesenchymal cells are key contributors and regulators of extracellular matrix, both in tissue morphogenesis, homeostasis and disease. Recent studies using single-cell transcriptomic analysis have demonstrated the transcriptomic heterogeneity of the lung mesenchyme and revealed the existence of novel mesenchymal cell populations in lung tissue^1,2^. This is of particular interest in pulmonary fibrosis, a disease characterized by excessive and uncontrolled mesenchymal proliferation and matrix deposition^3^. Evidence suggests that specific mesenchymal subsets may be the source of these pathological fibrinogenic cells in disease^4–8^. To understand the cellular and molecular mechanisms that interfere with repair and regeneration in disease, it is crucial to first define and isolate functionally distinct mesenchymal subsets in the normal lung.

The field is still struggling to decipher the cellular heterogeneity due to a lack of specific surface markers that identify functionally distinct mesenchymal populations. Different mesenchymal cell types have been described using a large variety of terminology and marker combinations, however there is a large overlap in surface marker profile making them difficult to isolate and study. This is partly due to the great level of plasticity of mesenchymal cells depending on microenvironmental cues^9,10^. It is therefore not surprising that some markers, originally identified in in vitro cultures, are differently expressed on uncultured primary cell populations^11,12^. These culture-induced changes are likely to affect cellular function and behavior and obscure true heterogeneity of native mesenchymal cells. To uncover specific cellular functions within this heterogeneous cell pool, novel markers are therefore needed that further separate primary, uncultured mesenchymal cell populations.

We have previously described a lung-resident population of CD90^+^CD105^+^ cells that give rise to fibroblast-like colonies in vitro^13^. Yet, the heterogeneity and native identity of these cells remain unclear, motivating further studies to uncover the cellular complexity of lung mesenchyme. Therefore, we aimed to identify novel markers for identification and prospective isolation of native mesenchymal lung populations. We identified CD13/aminopeptidase N as a marker that distinguishes two native populations of clonogenic mesenchymal cells with different proliferative potential that give rise to cells with fibroblastic morphology. Using bulk and single cell transcriptomic analysis together with RNA in situ hybridization, we were further able to identify that CD105^+^CD90^+^CD13^+^ clonogenic mesenchymal cells originate from a subset of adventitial fibroblasts.

## Results

### CD13 expression distinguishes two distinct CD105^+^CD90^+^ mesenchymal cell populations

Lung-resident mesenchymal cells are heterogeneous and the lack of specific surface markers limits the possibility to isolate and understand putative mesenchymal subsets. To improve prospective isolation of native uncultured mesenchymal subsets, we initially searched for marker candidates by examining the cell-surface proteome of culture expanded mesenchymal cells by mass spectrometry. Human lung-derived mesenchymal cells were isolated and expanded using two different protocols to acquire different phenotypes. Mesenchymal cells were cultured from tissue explants in DMEM supplemented with 10% fetal bovine serum (FBS, protocol 1) or from enzymatically generated single cell suspensions in StemMACS MSC Expansion media (protocol 2). Mass spectrometry and flow cytometry analysis was used to identify candidate cell-surface markers (Supplementary Fig. S1). Out of the 50 differentially expressed proteins identified in the mass spectrometry analysis we identified 13 cell-surface protein with differential expression between protocol 1 and 2 (Supplementary Fig. S1). Using flow cytometry we could validate differential expression at cell-surface level for three proteins (CD10, CD13, and CD26, Supplementary Fig. S1).

**Figure 1:**
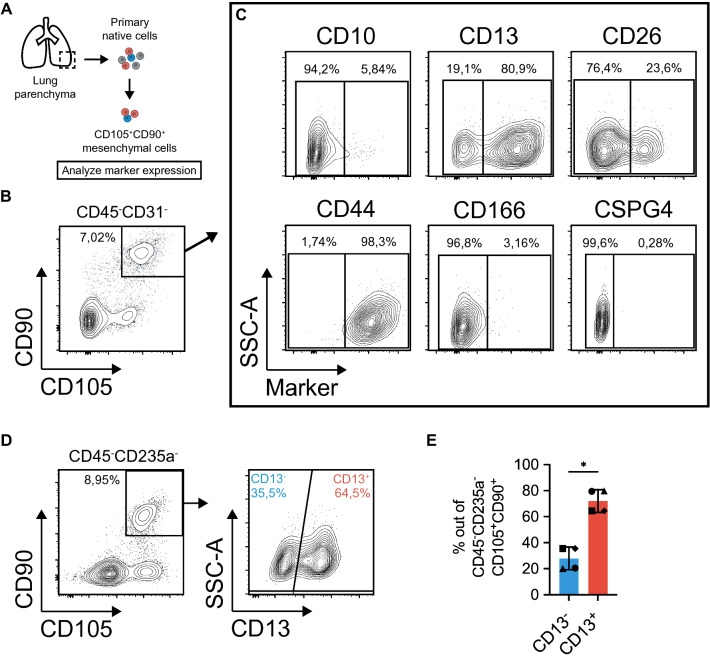
Flow cytometry analysis of candidate mesenchymal markers identifies distinct CD105^+^CD90^+^ populations based on CD13 expression. (**A**) Schematic of experimental design for analysis of surface marker candidates in primary CD105^+^CD90^+^ mesenchymal cells from human lung parenchyma. (**B**) Multicolor flow cytometry analysis showing gating of CD105^+^CD90^+^ mesenchymal cells in freshly isolated cells. The plot is shown after forward-scatter/side-scatter gating, exclusion of dead cells (7AAD), hematopoietic cells (CD45) and endothelial cells (CD31), and doublet exclusion (area versus height in forward-scatter). (**C**) Plots show candidate marker expression (x-axis) versus side-scatter (y-axis) in CD90^+^CD105^+^ mesenchymal cells. For markers CD10, CD13, and CD26, gates are set to define separated population based on marker expression profile. For markers CD44, CD166 and CSPG4, gates are set based on fluorescence minus one (FMO) controls. (**D**) Plots demonstrate gating for identification of CD105^+^CD90^+^CD13^−^ and CD105^+^CD90^+^CD13^+^ cells in cryopreserved primary/native cells from human lung parenchyma. The plots are shown after forward-scatter/side-scatter gating, exclusion of dead cells (7AAD), red blood cells (CD235a), and hematopoietic cells (CD45). Percentage of cells in each gate out of all cells in the plot, is indicated next to the gates. (**E**) Frequency of CD13^−^ and CD13^+^ cells in CD105^+^CD90^+^ mesenchymal cell population from healthy parenchymal lung tissue (n = 4). Bars represent mean (with SD). Paired two-tailed t-test was used for statistical analysis of data (*p < 0.05). *SSC-A* side scatter-area.

Next, we analyzed the marker expression on primary CD45^−^CD31^−^CD105^+^CD90^+^ mesenchymal cells from lung parenchyma, a population previously shown to enrich for fibroblast-like colonies, CFU-f^13^, aiming to identify novel markers to identify subpopulations of native lung mesenchymal cells (Fig. 1A,B). Interestingly, flow cytometry analysis demonstrated the presence of potential subpopulations of CD105^+^CD90^+^ mesenchymal cells when cells were examined for expression of CD10, CD13 and CD26 (Fig. 1C, Supplementary Fig. S2). On the other hand, the common mesenchymal markers CD44 and CD166 as identified in the mass spectrometry data (Supplementary Fig. S1), as well as the pericyte marker CSPG4 showed a homogeneous expression profile in CD105^+^CD90^+^ mesenchymal cells (Fig. 1C). Here, CD105^+^CD90^+^ mesenchymal cells clearly expressed high levels of CD44 while expression of CD166 and CSPG4 was low or absent (Fig. 1C).

As CD13 expression identified the most distinct subpopulations in all subjects in the initial screening (Fig. 1C; Supplementary Fig. S2), this marker was selected for further investigation. By analyzing the CD13 expression in cryopreserved primary CD45^−^CD235a^−^CD105^+^CD90^+^ mesenchymal cells from additional healthy donors (n = 4), we could confirm the existence of two distinct CD105^+^CD90^+^ mesenchymal cell populations defined as CD105^+^CD90^+^CD13^−^ and CD105^+^CD90^+^CD13^+^ in human lung parenchyma (Fig. 1D,E). The populations showed a similar frequency distribution across donors with CD13^+^ cells constituting the largest fraction of CD45^−^CD235a^−^CD105^+^CD90^+^ cells (Fig. 1E).

### Clonogenic fibroblast-like cells originate from both CD105^+^CD90^+^CD13^−^and CD105^+^CD90^+^CD13^+^ mesenchymal cell populations

**Figure 2:**
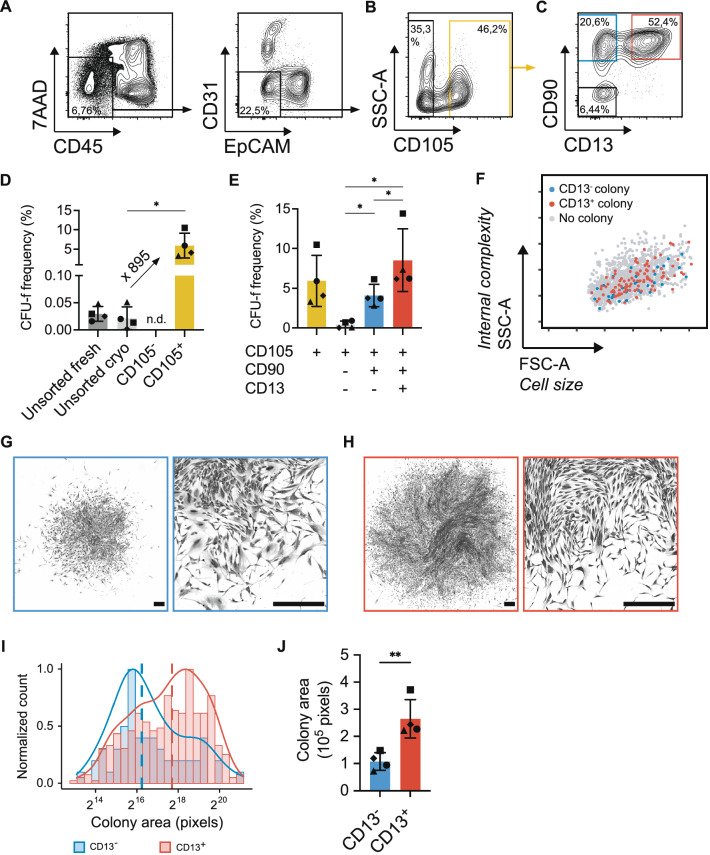
Clonogenic fibroblast-like cells originate from both CD105^+^CD90^+^CD13^−^ and CD105^+^CD90^+^CD13^+^ populations. (**A**–**C**) Gating strategy for sorting of mesenchymal cell populations for CFU-f assay. (**A**) Exclusion of dead cells (7AAD^+^) and non-mesenchymal lineage (lin^−^) cells (hematopoietic cells: CD45^+^, endothelial cells: CD31^+^, epithelial cells: EpCAM^+^) after forward-scatter/side-scatter gating. (**B**) Gates set for sorting of lin^−^CD105^−^ (black) and lin^−^CD105^+^ (yellow) cells. (**C**) Gates set for sorting of lin^−^CD105^+^CD90^−^CD13^−^ (black), lin^−^CD105^+^CD90^+^CD13^−^ (blue) and lin^−^CD105^+^CD90^+^CD13^+^ (red) cells. (**D**) CFU-f frequency in sorted lin^−^CD105^+^ cells compared to lin^−^CD105^−^ and unsorted lung cells (fresh and cryopreserved). (**E**) CFU-f frequency in sorted lin^−^CD105^+^CD90^−^CD13^−^, lin^−^CD105^+^CD90^+^CD13^−^ and lin^−^CD105^+^CD90^+^CD13^+^ cell populations, with lin^−^CD105^+^ cells as comparison. CFU-f frequency data (healthy donors, n = 3; non-tumorous lung cancer tissue, n = 1) is presented as mean (with SD). One-way ANOVA with Geisser–Greenhouse correction and Holm–Sidak correction for multiple comparisons was used for statistical analysis (*p < 0.05). (**F**) The plot shows forward-scatter and side-scatter characteristics for 1532 index-sorted cells (749 CD13^−^ and 783 CD13^+^) combined from 4 healthy donors. The 35 CD13^−^ cells (blue dots) and 97 CD13^+^ cells (red dots) formed colonies in CFU-f assays. Sorted cells that did not generate colonies are represented by grey dots. (**G**,**H**) Images of crystal violet stained colonies derived from CD13^−^ cells (**G**) and CD13^+^ cells (**H**). Scale bars represents 500 µm. (**I**) Representative histograms showing the distribution of colony size in CD13^−^ and CD13^+^ derived CFU-fs (crystal violet stained) from one healthy donor. Normalized colony count is shown on the y-axis and colony size is on the x-axis. Solid lines represent density plots. Vertical dashed lines represent median colony area. (**J**) The median colony size of CD13^−^ and CD13^+^ derived CFU-fs (healthy donors, n = 3; non-tumorous lung cancer tissue, n = 1). Bars represent mean (with SD) and dots represent median for individual subjects. Paired two-tailed t-test was used for statistical analysis of colony area data (**p < 0.01). *FSC-A* forward scatter-area, *SSC-A* side scatter-area, *CFU-f* colony forming unit-fibroblast.

To determine if the identified populations would enrich for cells with high proliferative potential (colony-forming cells), CD45^−^CD31^−^EpCAM^−^ (lin^−^) mesenchymal cells were sorted based on CD105, CD90 and CD13 expression (Fig. 2A–C) and cultured at clonal density to determine their CFU-f frequency (i.e. clonogenicity). First, we could confirm that clonogenic cells were highly enriched in the lin^−^CD105^+^ lung mesenchymal cell fraction (Fig. 2B), with a mean CFU-f frequency of 5.9% (range 3.5–7.2, Fig. 2D). Furthermore, a donor-dependent CFU-f enrichment with a mean enrichment of 895-fold (range 64.6–2435) in the lin^−^CD105^+^ fraction compared to unsorted cryopreserved lung cells was observed. In contrast, no colonies could be detected in the lin^−^CD105^−^ fraction (Fig. 2D). Based on the expression of CD90 and CD13, three major subsets could be separated in the lin^−^CD105^+^ fraction: CD90^−^CD13^−^, CD90^+^CD13^−^ and CD90^+^CD13^+^ (Fig. 2C). Notably, clonogenic cells were found to originate from both CD90^+^CD13^−^ (mean 4.1%, range 2.8–6.1) and CD90^+^CD13^+^ cell populations (mean 8.5%, range 6.2–14.4), with CFU-f frequencies being highest in the CD13^+^ cells. In contrast, cells from the CD105^+^CD90^−^ population had a very low CFU-f frequency (mean 0.4%, range 0–0.9, Fig. 2E). Moreover, the clonogenic capacity of CD105^+^CD90^+^CD13^−^ and CD105^+^CD90^+^CD13^+^ mesenchymal cells were confirmed by index sorting of one cell per well in 96-well plates (Supplementary Fig. S3). Retrospective identification of the flow cytometry profile of 1532 single cells using index sorting revealed that clonogenic cells in both CD105^+^CD90^+^CD13^−^ and CD105^+^CD90^+^CD13^+^ populations shared the same characteristics in terms of cell size and internal complexity, as reflected in forward scatter and side scatter characteristics, respectively (Fig. 2F). Both lin^−^CD105^+^CD90^+^CD13^−^ and lin^−^CD105^+^CD90^+^CD13^+^ cells displayed a characteristic fibroblastic morphology in culture (Fig. 2G,H). Taken together, these data suggest that fibroblast-like mesenchymal clonogenic cells originate from two cell populations in lung defined by their CD13 expression, and that native lung-resident mesenchymal cells are heterogeneous.

### CD13^+^ cells form larger colonies compared to CD13^−^ cells

To examine functional differences between CD105^+^CD90^+^CD13^−^ and CD105^+^CD90^+^CD13^+^ clonogenic mesenchymal cells, we first measured the cell-covered area of stained colonies as an estimation of cell number per colony. The analysis showed that although there was a wide distribution of colony sizes within both populations, the colony area was significantly larger in CD105^+^CD90^+^CD13^+^ derived colonies compared to CD105^+^CD90^+^CD13^−^ derived colonies (p = 0.0061, Fig. 2I,J).

### Native CD13^−^ cells upregulate CD13 surface marker expression upon in vitro culture

**Figure 3:**
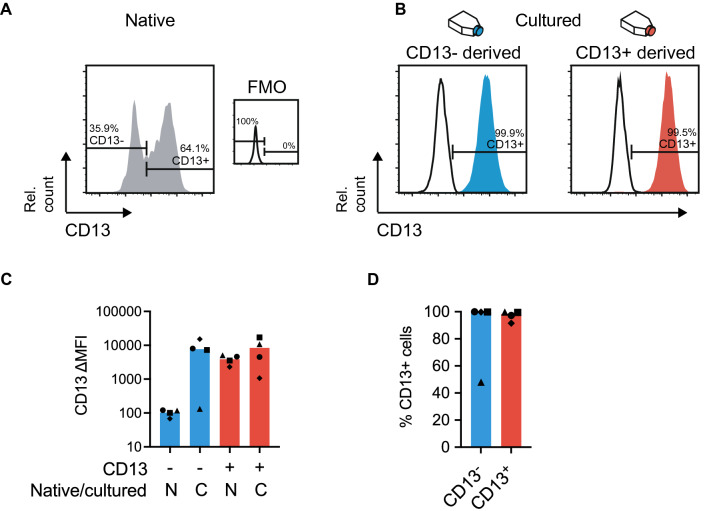
Native CD13^−^ cells upregulate CD13 expression in culture. (**A**) Representative histograms displaying cell-surface expression of CD13 as measured by flow cytometry on native CD105^+^CD90^+^CD13^−^ and CD105^+^CD90^+^CD13^+^ populations compared to negative (FMO) control. The plots show representative data after forward-scatter/side-scatter gating, exclusion of dead cells (7AAD^+^) and non-mesenchymal lineage cells (hematopoietic cells: CD45^+^, endothelial cells: CD31^+^, epithelial cells: EpCAM^+^). (**B**) Representative histograms showing cell-surface expression of CD13 in clonal cultures from sorted CD105^+^CD90^+^CD13^−^ and CD105^+^CD90^+^CD13^+^ populations (passage 4 bulk). Left plot show cells expanded from a CD105^+^CD90^+^CD13^−^ cell and right plot show cells expanded from a CD105^+^CD90^+^CD13^+^ cell. The percentage of cells in each gate out of all cells in the plot, is indicated next to the gates. Black lines represent unstained controls for culture expanded populations. Plots show data after forward-scatter/side-scatter gating and doublet exclusion (area versus height in forward-scatter). (**C**) Plot shows delta median fluorescence intensity (∆MFI) for CD13 between sample and control (FMO or unstained) on native (N) and cultured (**C**, passage 3–4) cells. (**D**) Plot displaying the percentage of CD13 positive cells in clonal cultures from sorted CD105^+^CD90^+^CD13^−^ and CD105^+^CD90^+^CD13^+^ populations (passage 3–4). Presented data is from healthy donor lungs (n = 4) and bars represent median values. *FMO* fluorescence minus one, *Rel. count* relative cell count (normalized to mode), *N* native, *C* cultured.

Prospective cell isolation by FACS allows for the identification of native cell phenotypes. However, subsequent in vitro cell culture is commonly associated with changes in cell properties which can result in cell differentiation and altered phenotype. To investigate if the identified native phenotypes of clonogenic mesenchymal populations would persist in culture, we examined the expression of CD13 on in vitro culture expanded cell clones derived from CD105^+^CD90^+^CD13^−^ and CD105^+^CD90^+^CD13^+^ cell populations (Fig. 3A,B). Interestingly, three out of four CD105^+^CD90^+^CD13^−^ derived clones showed significant upregulation of CD13 to an expression level similar to native and cultured CD105^+^CD90^+^CD13^+^ cells (Fig. 3C). The percentage of CD13 positive cells in clonal cultures were close to 100% for both CD105^+^CD90^+^CD13^−^ and CD105^+^CD90^+^CD13^+^ derived cells, with the exception for the one CD105^+^CD90^+^CD13^−^ derived clone with a lower average CD13 expression, with roughly 50% positive cells (Fig. 3D). Collectively, these results show that CD105^+^CD90^+^CD13^−^ cells can change their phenotype by upregulating CD13 when cultured in vitro on normal tissue culture plastic, which may also reflect functional alterations compared to the native cell.

### Transcriptomic analysis reveal an adventitial origin of CD13^+^ mesenchymal cells

**Figure 4:**
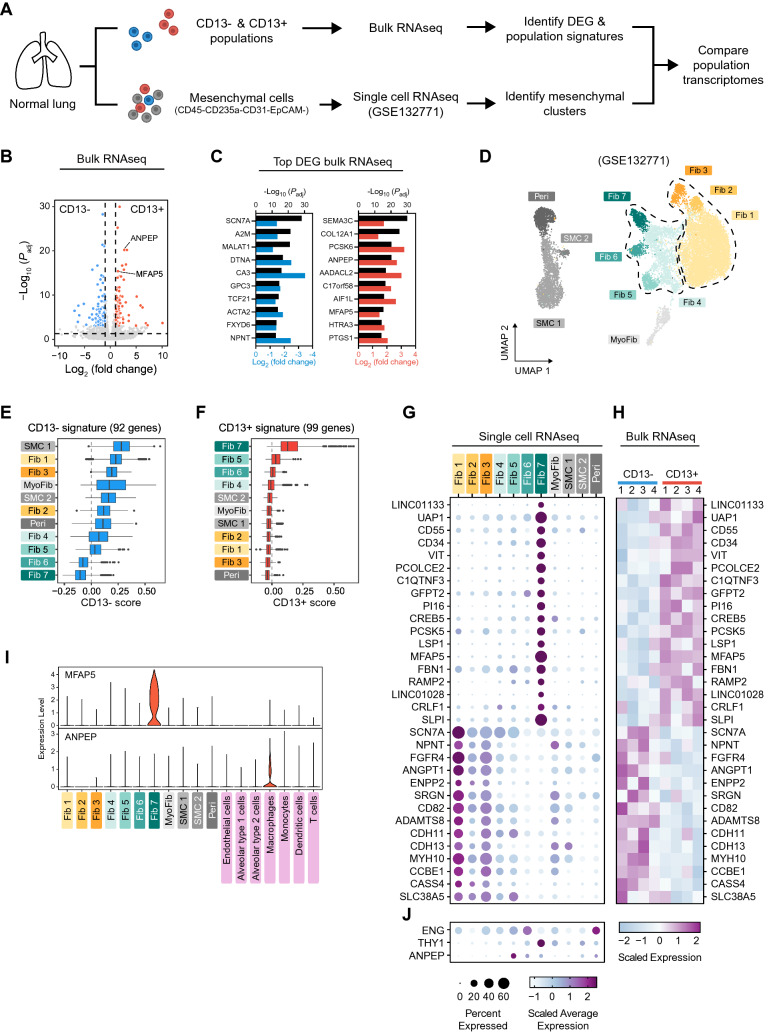
Transcriptomic analysis of CD13^−^ and CD13^+^ cells and cells in the lung mesenchymal compartment. (**A**) Schematic overview of transcriptomic analysis of uncultured CD13^−^ and CD13^+^ mesenchymal cell populations (bulk RNA-seq) and the lung mesenchymal compartment (single cell RNA-seq). (**B**) Volcano plot of bulk RNA-seq data showing differentially expressed genes between sorted CD13^−^ and CD13^+^ (CD45^−^CD235a^−^CD105^+^CD90^+^) cells (n = 4). Blue dots represent genes upregulated in CD13^−^ cells (CD13^−^ signature; adj. p < 0.001, Log2 FC < − 1) and red dots represent genes upregulated in CD13^+^ cells (CD13^+^ signature; adj. p < 0.001, Log2 FC > 1). (**C**) Top 10 (ranked by lowest adj. p value) enriched genes in CD13^−^ (left) and CD13^+^ (right) cells. Upper x-axis represent adj. p value (− log10 transformed, black bars) and lower x-axis represent fold change (log2 transformed, blue and red bars). (**D**) UMAP plot showing unsupervised clustering of the transcriptomes of 14,802 mesenchymal cells from 3 normal lungs analyzed by single cell RNA-seq (reanalysis of GSE132771 dataset from Tsukui et al.). The dashed lines separate fibroblast sub-clusters into alveolar and adventitial fibroblasts. (**E**,**F**) Boxplots showing the distribution of CD13^−^ and CD13^+^ gene signature scores for single cells in the different mesenchymal clusters identified in **D**. (**G**,**H**) Expression of 18 overlapping enriched genes between adventitial fibroblast cluster Fib 7 and CD13^+^ population and of 14 overlapping enriched genes between alveolar fibroblast cluster Fib 1 and CD13^−^ population in mesenchymal clusters (single cell RNA-seq, **G** upper panel) and CD13^−^/CD13^+^ populations (bulk RNA-seq, **H**). The lower panel in G shows the expression of *ENG*, *THY1* and *ANPEP* in mesenchymal clusters. Average gene expression in single cell data is presented in a dot plot in (**G**) where color represent average expression and dot size represent percentage of expressing cells within each cluster. Gene expression in bulk populations is presented as a heatmap in (**H**) with one column for each individual sample (n = 4). (**I**) Violin plots showing the expression of *MFAP5* and *ANPEP* in mesenchymal and non-mesenchymal cell clusters. (**J**) Expression of ENG, THY1 and ANPEP in mesenchymal clusters. *DEG* differentially expressed genes, *SMC* smooth muscle cells, *Peri* pericytes, *MyoFib* myofibroblast-like cells, *Fib* fibroblasts.

To further investigate the identity of native CD105^+^CD90^+^CD13^−^ and CD105^+^CD90^+^CD13^+^ clonogenic mesenchymal cells, the bulk transcriptomes of CD13^−^ and CD13^+^ populations were compared with the transcriptomes of single cells in the lung mesenchymal compartment (CD45^−^CD235a^−^CD31^−^EpCAM^−^ sorted cells from three normal lungs) from a published single cell RNA-seq dataset (GSE132771, Tsukui et al.^2^) (Fig. 4A). Transcriptomic analysis of sorted native CD45^−^CD235a^−^CD105^+^CD90^+^CD13^−^ and CD45^−^CD235a^−^CD105^+^CD90^+^CD13^+^ cells revealed 821 differentially expressed genes (DEG, *P*_adj_ < 0.05) between CD13^−^ and CD13^+^ populations, 442 of which had a fold change above 2 (Fig. 4B). The top DEG were selected as population specific gene signatures consisting of 92 DEG enriched in CD13^−^ cells (*P*_adj_ < 0.001 and log_2_ FC < − 1) and 99 DEG enriched in CD13^+^ cells (*P*_adj_ < 0.001 and log_2_ FC > 1, Fig. 4B; Supplementary Table S3). The gene coding for CD13, *ANPEP*, was among the top 10 DEG enriched in CD13^+^ cells (Fig. 4C). Consistant with typical mesenchymal characteristics, gene ontology enrichment analysis of the top DEG (CD13^−^ and CD13^+^ signatures) revealed extracellular matrix organization and extracellular matrix structural constituent to be the most enriched biological process and molecular function, respectively (Supplementary Fig. S4A). This suggests that differences in extracellular matrix related properties could be connected to CD13 expression. Matrisome genes among the signature genes are shown in Supplementary Fig. S4B according to their matrisome categories.

Analysis of mesenchymal single cell RNA-seq data revealed 4 primary cell groups consisting of 11 distinct cell clusters representing smooth muscle cells (SMC 1 and 2), pericytes (Peri), myofibroblast-like cells (MyoFib), and fibroblasts (Fib 1, 2, 3, 4, 5, 6 and 7) as depicted in Fig. 4D. Annotation of mesenchymal cell populations was based on the expression of canonical and recently published markers (Supplementary Fig. S5). To identify single cell clusters that could represent CD105^+^CD90^+^CD13^−^ and CD105^+^CD90^+^CD13^+^ cell populations, scores were calculated for each cell based on the bulk gene signatures for CD13^−^ and CD13^+^ populations (Fig. 4E,F). The gene signature scoring revealed high transcriptomic similarity between adventitial fibroblast cluster Fib 7 and the CD105^+^CD90^+^CD13^+^ cell population, whereas both SMC cluster (SMC 1) and alveolar fibroblast clusters (Fib 1 and 3) showed the highest similarity to the CD105^+^CD90^+^CD13^−^ cell population. In addition, comparing the overlap between population gene signatures and DEG in single cell RNA-seq data showed that the majority of CD13^+^ enriched genes were found in adventitial fibroblast cluster Fib 7 (18 out of 26 genes) whereas the majority of CD13^−^ enriched genes were present in alveolar fibroblast cluster Fib 1 (14 out of 24 genes, Supplementary Fig. S6). Side-by-side comparison of the expression of these genes in bulk and single cells RNA-seq data highlighted the transcriptomic similarity between CD13^+^ cells and adventitial fibroblasts and CD13^−^ cells and alveolar fibroblasts (Fig. 4G,H).

Next, we aimed to identify a suitable marker with high specificity for the identified CD13^+^ adventitial population in order to validate the spatial localization in lung using in situ hybridization. Expression of *THY1*, *ENG* and *ANPEP* (genes coding for CD90, CD105 and CD13) was detected in several clusters, yet expression was more abundant in adventitial fibroblast clusters than alveolar fibroblast clusters (Fig. 4J). Interestingly, *MFAP5* was among the top 10 enriched genes in the CD13^+^ population (Fig. 4C) and it was also shown to be highly abundant and specific for the Fib 7 cluster (Fig. 4G). Importantly, when comparing the expression of *MFAP5* in mesenchymal cells with other lung cell types identified among CD235a^−^ sorted cells (Supplementary Fig. S7), *MFAP5* expression remained specific to Fib 7 and was low in endothelial cells, epithelial cells (alveolar type 1 and type 2 cells), macrophages, monocytes, dendritic cells, and T cells (Fig. 4I). In comparison, *ANPEP* (CD13) expression was most abundant in macrophages and not specific for Fib 7 (Fig. 4I).

### MFAP5^+^ fibroblasts are localized to vascular adventitia

**Figure 5:**
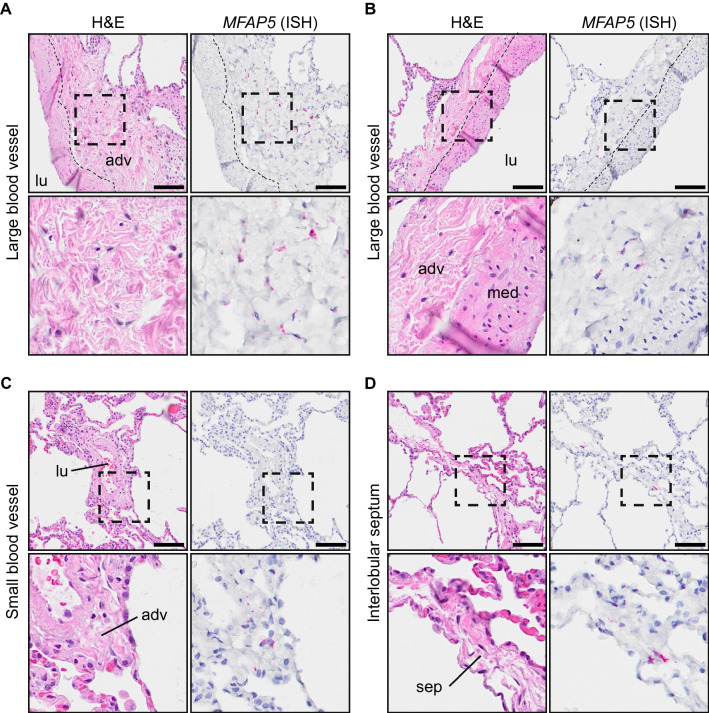
Identification of *MFAP5*^+^ cells in human distal lung tissue. (**A**–**D**) Images of serial sections of normal human lungs stained with hematoxylin and eosin (H&E, left panel) and probed by RNA in situ hybridization for *MFAP5* (right panel). The scale bar is 100 µm. Areas inside rectangles (dashed) are magnified in lower panels. All images in the figure are from the same individual. *MFAP5*^+^ cells in adventitia of larger blood vessels (**A**,**B**), small blood vessel (**C**) and interlobular septum (**D**). *adv* adventitia, *med* media, *lu* lumen, *sep* interlobular septum.

To validate the adventitial identity of CD13^+^ cells as suggested by the transcriptomic analysis, we next performed RNA in situ hybridization for *MFAP5* in healthy distal lung tissue sections. In accordance with the transcriptomic signature, *MFAP5*^+^ cells were found within the adventitia of large and small blood vessels as well as in the loose connective tissue of interlobular septa, which contain veins and lymphatic vessels (Fig. 5, Supplementary Fig. S8A–I). Occasionally, *MFAP5*^+^ cells were found in the loose connective tissue of the visceral pleura which is continuous with interlobular septa (Supplementary Fig S8J,K). Importantly, *MFAP5* expression was not detected in SMC of the tunica media or in alveolar tissue (Supplementary Fig. S9).

## Discussion

We describe the isolation and enrichment of a subset of adventitial mesenchymal cells with clonogenic potential based on CD105^+^CD90^+^CD13^+^ expression. We observed that this population had an increased frequency of clonogenic cells and showed an increased proliferative potential compared to CD105^+^CD90^+^ mesenchymal cells which lacked CD13 expression, suggesting functional differences between these phenotypes. CD13, also known as aminopeptidase N, is a membrane bound metalloprotease with multiple reported functions, including enzymatic regulation of peptides, migration, chemotaxis, proliferation, adhesion, and angiogenesis^14–16^. It is unclear which mechanistic role CD13 plays in the identified CD13^+^ cells. However, the upregulation of CD13 on CD105^+^CD90^+^CD13^−^ cells that attach and proliferate in culture could indicate a mechanistic link between CD13 and cell adhesion or proliferation in these cells, however, this needs to be further studied.

Through the use of transcriptomic analysis we aimed to uncover the origin of clonogenic mesenchymal cells in human lung and to describe the in vivo identity of CD105^+^CD90^+^CD13^−^ and CD105^+^CD90^+^CD13^+^ mesenchymal cells. Based on transcriptomic similarity, we could observe that a distinct subset of adventitial fibroblasts within the mesenchymal compartment resemble CD105^+^CD90^+^CD13^+^ mesenchymal cells and constitute a potential major source of clonogenic mesenchymal cells in lung. This fibroblast sub-cluster (Fib 7) expressed several genes previously reported in a fibroblast population located in vascular adventitia (adventitial fibroblasts) as determined by in situ hybridization assays^2,17^. Interestingly, this cluster was clearly distinct from classical CD146^+^ pericytes (Peri cluster), which have been a proposed source of self-renewing mesenchymal progenitor cells from multiple tissues^18,19^. Our results agree with previous work by Corselli et al., showing that CD34^+^CD146^−^ adventitial cells from the tunica adventitia of arteries and veins in human tissues, including fetal lung tissue, is a source of clonogenic mesenchymal cells^20^. The notion that adventitial fibroblasts may serve as fibroblast progenitors is supported by Buechler and collegues who performed an integrative analysis with single cell RNA-seq datasets of fibroblasts from multiple tissues^21^. They observed two universal fibroblast populations identified in all tissues analyzed, one of which had an transcriptomic identity similar to adventitial fibroblasts and defined by *PI16* expression, a gene enriched in CD105^+^CD90^+^CD13^+^ cells and in the Fib 7 cluster.

We could indirectly confirm the adventitial localization of CD105^+^CD90^+^CD13^+^/Fib 7 cells through RNA in situ hybridization analysis for *MFAP5*, the gene coding for microfibrillar-associated glycoprotein 2 (MAGP2) which is a component of elastic fibers and microfibrils^22^. The tunica adventitia has been described to be a part of a previously unrecognized fluid-filled pre-lymphatic interstitial space^23^. In this space, well-organized collagen bundles form a reticular-like pattern, similar to that of lymphatic tissue. In lymph nodes, fibroblastic reticular cells form collagen-matrix containing fibers that build up a network of microchannels (conduits)^24^. Interestingly, Barron and colleagues observed that MFAP5 was a component of conduits in human spleen, lymph node, and tonsil^25^. They also showed that VCAM1^+^ fibroblastic reticular cells wrapped these MFAP5^+^ conduits. Furthermore, they could identify MFAP5^+^ perivascular adventitial elastic fibers in human skin. They could observe that fibroblasts occasionally associated with these MFAP5^+^ structures. We identified several genes related to elastic fiber formation to be upregulated in CD13^+^ cells (*MFAP5*, *BMP7*, *FBN1*, *FBLN2* and *EMILIN2*). Elastic fibers can be found throughout the lung, including the alveoli and the tunica media of vessels. Yet, the spatial distribution of *MFAP5*^+^ cells was limited to the tunica adventitia and similar regions containing loose connective tissue, suggesting a specific role of MFAP5 in these structures. Importantly, in relation to fibrotic lung disease, microfibrils serve as reservoirs for transforming growth factor β (TGF-β) and MFAP5 can bind both TGF-β and bone morphogenic protein (BMP) growth factors^26^.

The idea that these structures could serve as pre-lymphatic channels put CD105^+^CD90^+^CD13^+^ mesenchymal cells in a convenient spatial position to interact with immune cells and both respond to and deliver solube mediators. Indeed, the adventitia has been described as an immunologically active site and proposed to serve as a “regional hub for tissue immunity”^27,28^. Fibroblast-like adventitial stromal cells in bronchovascular cuffs have been shown to provide a regulatory niche for group 2 innate lymphoid cells in mice^28^. These stromal cells expressed *IL33* which was also upregulated in Fib 7 cluster (Supplementary Fig. S6D). Interestingly, several secreted factors were upregulated in CD105^+^CD90^+^CD13^+^ cells, further highlighting a potential immunoregulatory function (Supplementary Fig. S4B).

Contrary to CD105^+^CD90^+^CD13^+^ cells, CD105^+^CD90^+^CD13^−^ mesenchymal cells appeared to show a more diverse transcriptomic profile, similar to alveolar fibroblasts, pericytes, SMC and myofibroblast-like cells. This could indicate that the sorted CD105^+^CD90^+^CD13^−^ population is heterogenous and contains more than one cell type. Clearly, these findings warrant further investigations into the identity of CD105^+^CD90^+^CD13^−^ clonogenic mesenchymal cells in lung. A previous study from our lab demonstrated that colony-forming mesenchymal cells in human lung are CD146^−^ contrary to what has been observed in other tissues, including bone marrow^13^. CD146 is highly expressed on pericytes and SMC. Therefore, it could be speculated that CD105^+^CD90^+^CD13^−^ clonogenic mesenchymal cells originate from alveolar fibroblasts (Fib 1, 2 and 3). However, this remains to be confirmed as a second source of CD105^+^CD90^+^ mesenchymal clonogenic cells.

Our study has several strengths. First, analyses were performed on cells from healthy adult human lungs. Secondly, we performed prospective isolation and characterization on uncultured native cells. A source of confusion and inconsistency in the literature is the use of in vitro defined phenotypes and markers to describe and anatomically localize native cell populations. However, the discrepancy between marker expression on native uncultured cells and cells expanded in vitro can be striking. Accordingly, we could observe that CD105^+^CD90^+^CD13^−^ cells upregulated CD13 expression after culture expansion (observed as early as passage 1), suggesting that CD13 expression is highly influenced by culture condition. Furthermore, we have observed that the commonly used pericyte marker CSPG4, is highly expressed on culture expanded mesenchymal cells, however uncultured CD105^+^CD90^+^ cells do not express CSPG4, suggesting that a pericyte-phenotype is acquired after culture expansion. One caveat is that no gain and loss of function experiments were performed. As such we do not know if inducing CD13 expression in CD13^−^ cells by in vitro culturing is accompanied with a functional switch of the cells. Similarly, no interaction studies with other cell types of interest including epithelial cells, endothelial cells and immune cells were performed.

In summary, we have identified an adventitial fibroblast subset as the origin for clonogenic mesenchymal cells in human lung. These clonogenic mesenchymal cells can be prospectively isolated from primary tissues by CD105^+^CD90^+^CD13^+^ expression. The transcriptomic analysis provided a further description of mesenchymal cell heterogeneity and the potential identity of mesenchymal progenitors in human lung. It remains to disentangle the continuum of mesenchymal cell phenotypes in their in situ niches in health and disease. With the identification of novel cell surface markers this could be realized and a deeper understanding of the mesenchymal cell phenotypes could be accomplished and subsequently addressed in fibrosing diseases.

## Materials and methods

### Patient description and ethical approval

Lung explants from healthy donors (n = 6) and from a patient with chronic rejection after lung transplantation (n = 1) were acquired from Sahlgrenska University Hospital in Gothenburg and Skåne University Hospital in Lund. The healthy donor lungs could be included in the study as no matched recipient could be identified for transplantation. Lung tumor resection tissue from one lung cancer patient without chronic obstructive pulmonary disease (n = 1) was acquired from Skåne University Hospital in Lund. Patient information is summarized in Supplementary Table S1. Written informed consent to participate in the study was obtained from all participants or from their closest relatives. No organs/tissues were procured from prisoners. The study was approved by the Swedish Research Ethical Committee in Lund (FEK 2015/891 and FEK 2006/91) and Gothenburg (FEK 657-12/2012 and FEK 2008/413) and all experimental protocols were carried out in accordance with guidelines approved by the ethical committees.

### Mass spectrometry analysis of cultured cells

Details on mass spectrometry analysis of cultured cells for cell-surface marker discovery are provided in the supplementary materials and methods section. The mass spectrometry proteomics data have been deposited to the ProteomeXchange Consortium via the PRIDE^29^ partner repository with the dataset identifier PXD029028.

### Isolation of primary lung cells

Parenchymal lung tissue from explants or non-tumorous lung cancer resections was dissected out, avoiding visible airways. Tissue pieces (< 30 mm^3^) were washed with Dulbecco’s phosphate-buffered saline (DPBS) and cut into small pieces (< 3 mm^3^) before being enzymatically digested using 100 U/mL DNase I (Sigma-Aldrich), 300 U/mL collagenase type 1 (Gibco) and 1 mg/mL hyaluronidase (Serva Electrophoresis) in DPBS for 1.5–2 h at 37 °C. Single-cell suspensions were separated from digested tissue by filtration through a 100 µm filter. Single-cell suspensions were incubated in red blood cell (RBC) lysis buffer (155 mM NH_4_CL, 10 mM KHCO_3_) for 5 min to lyse RBCs. An aliquot of the isolated primary lung cells was plated for colony forming unit-fibroblast (CFU-f) assay as described below and the remaining cells were immediately processed for flow cytometry or cryopreserved in StemMACS MSC Expansion media (Miltenyi Biotec) containing 1% antibiotic–antimycotic (AB/AM) solution (Sigma-Aldrich) mixed 1:1 (v/v) with DPBS containing 15% DMSO, 50% FBS (Life Technologies) and 20 IU/mL Heparin (Leo Pharma).

### Flow cytometry and fluorescence-activated cell sorting (FACS) of native cells

Freshly isolated and cryopreserved lung cells were used for flow cytometry and cell sorting. Cryopreserved lung cells were thawed at 37 °C and diluted in StemMACS MSC Expansion medium supplemented with 1% AB/AM, 1 mM MgCl_2_ and 100 Kunitz units/mL DNase I (Sigma-Aldrich) before centrifugation and removal of supernatant containing DMSO. To block unspecific binding to Fc-receptors, cells were incubated in PBS containing 3.3 mg/mL human immunoglobulin (Gammanorm, Octapharma) and 0.1% FBS (Gibco) for 10 min at 4 °C. After blocking, cells were stained with directly conjugated antibodies (Supplementary Table S2) for 20 min at 4 °C. Prior to analysis, cells were stained with the viability dye 7-aminoactinomycin D (7AAD, 2.5–4.0 µg/mL, Sigma-Aldrich or eBioscience) and filtered through a 35 µm cell strainer. Stained cells were analyzed on a BD LSR Fortessa or on a BD FACS Aria IIu/III cell sorter fitted with a 100 µm nozzle (BD Bioscience). Sorted cells were collected in StemMACS MSC Expansion media for CFU-f assay and clonal expansion or in extraction buffer from the PicoPure RNA isolation kit for RNA isolation. Flow cytometry data was analyzed using FlowJo software version 10.7.1 (BD Bioscience). Data from index sorting was processed and analyzed using the flowCore and indexSort packages in R (version 3.6.1) with R Studio (version 1.2.1335).

### Flow cytometry analysis of cultured cells

Cultured mesenchymal cells were incubated in PBS containing 3.3 mg/mL human immunoglobulin (Gammanorm; Octapharma) and 0.1% FBS (Gibco) for 10 min at 4 °C, to block unspecific binding to Fc-receptors, followed by staining with directly conjugated antibodies for 20 min at 4 °C. For cultured cells antibodies against the following surface markers were used: CD90-PE (clone 5E10), CD105-APC (clone 266), CD73-PE (clone AD2), CD44-APC (clone G44-26), CD166-BV421 (clone 3A6), CD10-BV421 (clone HI10a), CD13-PE (clone WM15), CD26-APC (clone M-A261) and CSPG4-PE (clone 9.2.27; all from BD Bioscience). Stained cells were run on a BD LSR II cytometer (BD Bioscience) and analyzed using FlowJo software version 10.5.3 (BD Bioscience).

### CFU-f assay

CFU-f frequencies of unsorted primary lung cell suspensions (fresh and cryopreserved) and sorted (FACS) cell populations were determined as previously described^30^. For unsorted lung cell suspensions, cells were plated at 3.16 × 10^3^ and 4.21 × 10^3^ cells/cm^2^ in 6-well plates. A minimum of 57 000 cells per sample were plated for unsorted cells. For bulk sorted cell populations, cells were plated at a mean density of 27.62 cells/cm^2^ (range 23.53–51.38) in 6-well plates. Bulk sorted samples with a minimum of 200 plated cells (mean 1952, range 225–9138) were included in the analysis. For single cell (index) sorted cells, 1 cell per well was sorted in 96-well plates. A total of 749 CD13^−^ cells and 783 CD13^+^ cells were index sorted from 4 healthy donors with a minimum of 129 sorted cells per donor (mean 191.5, range 129–288). Cells were cultured in StemMACS MSC Expansion media (Miltenyi Biotec), containing 1% AB/AM, for 14 days (unsorted and bulk sort) or 21 days (single cell sort). Culture media was changed on day 3, 7, and 14. On the final day of culture, cells were washed with DPBS, fixated with 100% methanol (Sigma-Aldrich) and stained with 0.5% crystal violet dye (Merck, Darmstadt, Germany). CFU-f from unsorted cells and single cell sort were enumerated manually using an Olympus CKX41 inverted brightfield microscope (Olympus). CFU-f from single cells were either enumerated after crystal violet staining or directly in live cell cultures if used for culture expansion. For CFU-fs from bulk sorted cells, whole well images were first captured using a Nikon Eclipse Ti2 (Nikon) and CFU-f were enumerated manually with the cell counter plugin in ImageJ version 2.0.0-rc-69/1.52p. CFU-f were defined as colonies containing ≥ 40 fibroblast-like cells. Shading correction was performed on images used to visualize colony and cell morphology by dividing images with a flat-field (empty well) image in ImageJ.

### Colony area measurements

Colony areas of CFU-f were determined by measuring cell-covered area (in pixels) in microscopy images taken of crystal violet stained colonies. De-identified greyscale images of individual wells from CFU-f assay of bulk sorted populations were imported into ImageJ (version 2.0.0-rc-69/1.52p.) and made binary and inverted. Individual colonies were manually selected and the selected area and percentage of cell-covered area for each colony was measured. Noticeably damaged colonies and colonies that could not be well separated were not measured. Measurements were performed by two blinded evaluators and the average of the median colony size for each cell population was used for statistical analysis.

### Clonal expansion of CD13^−^ and CD13^+^ subsets

Individual colonies (CFU-f) generated from index sorted single cells per well in 96-well plates were passaged at day 21 after sorting and expanded in StemMACS MSC Expansion media, with media changed every 2–3 days.

### Bulk RNA sequencing and data analysis

Total RNA was isolated from bulk sorted CD45^−^CD235a^−^CD105^+^CD90^+^CD13^−^ and CD45^−^CD235a^−^CD105^+^CD90^+^CD13^+^ cells using Arcturus PicoPure RNA isolation kit according to the manufacturer’s instructions (Thermo Fisher Scientific). The quantity and integrity of RNA samples was measured using the Agilent 2100 Bioanalyzer system (Agilent technologies). RNA samples used for library preparation had an average RIN value of 8.125 (range 6.9–10). cDNA synthesis and mRNA library preparation were prepared from 0.45 to 1 ng of total RNA using SMART-Seq v4 Ultra Low Input RNA Kit for Sequencing (Takara Bio) and Nextera XT DNA Library Preparation Kit (Illumina). A total of 8 libraries were pooled and run across two lanes of a NovaSeq 6000 S2 flow cell (Reagent Kit v1.5, 200 cycles, Illumina) on a NovaSeq 6000 System (Illumina). Demultiplexed FASTQ files were generated using bcl2fastq software with default settings followed by quality control analyses of raw sequencing data using FastQC. Reads were aligned to the Human GRCh38 genome with STAR aligner and raw read counts were generated using featureCounts. Summary statistics were generated using multiQC.

### Bioinformatics analysis of bulk RNA-seq data

Data containing raw read counts were analyzed using the DESeq2 package in R^31^. Genes with a minimum of 1 CPM in at least two samples were kept for differential gene expression analysis. Testing for differentially expressed genes was performed in a paired manner, by formulating the design to test the effect of cell populations (CD13^−^ vs CD13^+^) while controlling for the effect of lung donor. The adjusted p value cutoff (false discovery rate) was set to 0.05 (5%).

### Analysis of publicly available single cell RNA-seq data

Single cell RNA-seq data from Tsukui et al. was obtained from the Gene Expression Omnibus (GSE132771)^2^. Processed data consisting of features (genes), cell barcodes and gene barcode matrix (containing expression counts) for CD45^−^CD235a^−^CD31^−^EpCAM^−^ sorted mesenchymal cells and CD45^−^ sorted cells (all lung cells) from three normal lungs (NML1, NML2 and NML3) were downloaded and analyzed using the Seurat package in R. First, low quality libraries and potential multiplets were removed from each sample by removing cells with number of features, number of counts or percentage mitochondrial genes five times the median absolute deviation above the median value. An initial analysis was performed using the Seurat v3 workflow with the Harmony package for sample integration, consisting of NormalizeData, FindVariableFeatures (selection.method = "vst", nfeatures = 2000), ScaleData (vars.to.regress = c("nCount_RNA","percent.mt")), RunPCA, RunHarmony (group.by.vars = "orig.ident"), RunUMAP (reduction = "harmony"), FindNeighbors (reduction = "harmony", dims = 1:30), FindClusters (resolution = 0.6). Default function settings were used if not mentioned otherwise. For the mesenchymal dataset, distinct clusters contaminating non-mesenchymal cells (endothelial cells, epithelial cells, immune cells and mesothelial cells) and multiplets were removed. The Seurat workflow (with harmony) was performed again on the filtered mesenchymal dataset with resolution set to 0.3 for the FindClusters function. The function FindAllMarkers (only.pos = TRUE) in the Seurat package was used to identify differentially expressed genes (p < 0.01, average logFC > 0.25, expressed in > 10% of cells). Gene signature scores for single cells were calculated using the AddModuleScore function in Seurat. The average expression levels of a signature gene set is subtracted by the average expression levels of a control gene set as described by Tirosh et al.^32^. Upregulated genes in the CD13^−^ population (*P*_adj_ < 0.001 and log_2_ FC < − 1) and CD13^+^ population (*P*_adj_ < 0.001 and log_2_ FC > 1) were selected as signature gene sets for CD13^−^ and CD13^+^ populations, respectively. To compare the expression of *MFAP5* and *ANPEP* in among mesenchymal clusters and non-mesenchymal clusters, the Seurat objects for the mesenchymal dataset and the dataset containing all lung cells were merged and expression counts were normalized. Only major non-mesenchymal clusters from the CD45^−^ cell dataset were identified and included in the comparison (Supplemental Fig. S7).

### RNA in situ hybridization

Formalin-fixed paraffin-embedded distal lung tissue from four healthy donors were sectioned (4 µm) and placed on Superfrost Plus glass slides (cat.no 631-9483, VWR). Slides were prepared for In situ hybridization using RNAscope 2.5 HD detection reagent RED (cat.no. 322360, ACD Bio-Techne) according to the manufacturer’s assay manual. In brief, slides were baked at 60 °C for 1 h, followed by deparaffinization and treatment with hydrogen peroxide for 10 min. Slides were boiled in RNAscope Target Retrieval solution (ACD Bio-Techne) for 15 min, washed and airdried overnight. Slides were treated with protease plus for 30 min at 40 °C followed by probe hybridization to MFAP5 (cat.no. 573791, ACD Bio-Techne), along with assay-included positive and negative control probes, for 2 h at 40 °C. Signal amplification was performed by consecutive incubations steps with Amp1–Amp6. RED-B and Red-A (1:60) solution was added for 10 min at RT for signal detection. Slides were counterstained with 50% Mayer’s hematoxylin solution and dipped in 0.02% Ammonia water. Slides were dipped in xylene, mounted with VectaMount and scanned the following day at 20× magnification (Olympus VS120-L100-FL080) and viewed with OlyVIA software 2.9 and QuPath software 0.3.

### Statistical analysis

For statistical analysis of flow cytometry and CFU-f data GraphPad Prism version 8.4.3 was used. Data are presented as mean (bar or horizontal line) with standard deviation (error bars), together with individual values (dots) if not stated otherwise. Statistical analysis of RNA-seq data was performed in R (version 4.0.3).

## Supplementary Information

Supplementary Information.

## Data Availability

Raw and processed RNA sequencing data generated in this study are deposited to the Gene Expression Omnibus repository under the accession number GSE173738 and will be available to the public after publication.

## Abbreviations

7AAD: 7-Aminoactinomycin D
AB/AM: Antibiotic/antimycotic
BMP: Bone morphogenic protein
CFU-f: Colony forming unit-fibroblast
CPM: Counts per million
DEG: Differentially expressed gene
DMSO: Dimethyl sulfoxide
DPBS: Dulbecco’s phosphate-buffered saline
FACS: Fluorescence-activated cell sorting
FBS: Fetal bovine serum
Fib: Fibroblast
MAGP2: Microfibrillar-associated glycoprotein 2
MyoFib: Myofibroblast-like cell
Peri: Pericyte
RBC: Red blood cell
SMC: Smooth muscle cell
TGF-β: Transforming growth factor β
