# Supplementary material for: CD105+CD90+CD13+ identifies a clonogenic subset of adventitial lung fibroblasts
Source: Sci Rep. 2021 Dec 24;11:24417. doi: 10.1038/s41598-021-03963-9 (PMC8709856; doi:10.1038/s41598-021-03963-9)

Supplementary information for

$CD105^+CD90^+CD13^+$ identifies a clonogenic subset of adventitial lung fibroblasts

Måns Kadefors, Sara Rolandsson Enes, Emma Åhrman, Barbora Michaliková, Anna Löfdahl, Göran Dellgren, Stefan Scheding, Gunilla Westergren-Thorsson

Supplementary Materials and Methods

Culture of human lung mesenchymal cells for cell-surface marker discovery

Primary human lung mesenchymal cells were isolated using two different protocols from airways and parenchymal lung tissue from healthy donor lungs (n=4) as previously described[1,2]. In protocol 1, cells were isolated using explant cultures and cultured in Dulbecco's modified eagle medium (DMEM) with high glucose (4.5 g/L, Gibco) supplemented with 10% fetal clone III (GE Healthcare Hyclone), 1% L-glutamine (Sigma-Aldrich), 1% amphotericin B (Gibco) and 0.5% gentamicin (Gibco) at 37°C and 10% CO_2. In protocol 2, single cell suspensions, generated by enzymatic digestion of tissue, were plated and cultured in StemMACS MSC Expansion media (Miltenyi Biotec) supplemented with 1% AB/AM solution (Sigma-Aldrich) at 37°C and 5% CO_2. All cells were kept at 70-90% confluence and passaged by detaching enzymatically with TrypLE Express (Gibco). Cells at passage 4-5 were used for experiments.

Sample preparation for data-dependent acquisition (DDA) mass spectrometry

Four human cell types were cultured for analysis by data-dependent acquisition (DDA) mass spectrometry (MS) and generation of a spectral library to quantify data-independent acquisition (DIA) data. The cells used included a human neural epithelial-like stem cell line (Sai2, passage

33, a gift from Dr Marie Jönsson), a human fetal lung fibroblast cell line (HFL1, passage 18), primary human aortic smooth muscle cells (HASMC, passage 11, a gift from Dr Karin Tran-Lundmark) and primary human lung microvascular endothelial cells (HMVEC-L, passage 3). Cell layers from cultured cells were harvested with 10 mM Hepes buffer (pH 8) containing 10 mM DTT and 4% SDS using a cell scraper. Collected cell layers were heated at 95°C for 5 minutes followed by sonication using a Bioruptor Plus (Diagenode, Liege, Belgium) for 10 minutes with cycles of 15 seconds on and 15 seconds off at high power. Protein content was determined using Pierce BSA Protein Assay Kit (Thermo Fisher Scientific, cat. no. 23225) according to manufacturer's instructions. A total of 100 µg protein was pooled from 2-3 wells for each cell type, resuspended in Laemmli buffer (1x, Bio-Rad Laboratories, Hercules, USA) containing 2.5% (v/v) β-mercaptoethanol and boiled at 95 °C for 5 minutes. Protein samples were separated on SDS-PAGE (4-15% Mini-PROTEAN® TGX Stain-Free Gel, Bio-Rad Laboratories) at 200 V. The gel was stained with GelCode Blue Safe Protein Stain (Thermo Fisher Scientific, Rockford, USA) and each lane was cut into 45 bands that were pooled into 10 fractions and in-gel digestion was performed as previously described[3].

Sample preparation for data-independent acquisition (DIA) mass spectrometry

Cell layers from cultured mesenchymal cells were harvested with 10 mM Hepes buffer (pH 8) containing 10 mM DTT and 4% SDS using a cell scraper. Collected cell layers were heated at 95°C for 5 minutes followed by sonication using a Bioruptor Plus (Diagenode) for 10 minutes with cycles of 15 seconds on and 15 seconds off at high power. Cell lysates were alkylated with 50 mM iodoacetamide (IAA, Sigma) for 45 minutes at room temperature. To generate tryptic peptides and remove contaminants Single-Pot Solid-Phase-enhanced Sample Preparation (SP3) was performed utilizing paramagnetic beads (Thermo Fisher Sera-Mag Speed Beads A and B; Sigma-Aldrich)[4]. Briefly, protein samples were first dried and resuspended in liquid

chromatography-grade water, then incubated with beads in 50% acetonitrile (ACN) containing 0.17% formic acid (FA) for 10 minutes to bind proteins to beads. Placed on a magnet, bead-bound proteins were washed with 70% ethanol and then with 100% ACN. Off the magnet, tryptic digestion was performed on the immobilized proteins by incubating in 100 mM ammonium bicarbonate (Sigma-Aldrich) containing 250 ng/µL trypsin (sequencing grade modified trypsin porcine; Promega, Madison, USA) overnight (16 h) at 37°C. The peptide-bead mixture was then diluted with ACN to 95% ACN and incubated for 10 minutes. Placed on the magnet again, the bead-bound peptides were washed with 100% ACN. Off the magnet, the peptides were eluted in 2% DMSO in water and mixed 1:1 (v/v) with a buffer containing 4% ACN, 0.4% FA and iRT peptides (1:10 v/v; Biognosys, Schlieren, Switzerland).

LC-MS/MS analysis

Liquid chromatography-tandem mass spectrometry (LC-MS/MS) analysis was performed on a Q-Exactive Plus mass spectrometer (Thermo Fisher Scientific). Peptide separation was carried out by an EASY-nLC 1000 liquid chromatography system (Thermo Fisher Scientific) connected to an RP-HPLC EasySpray column (ID 75 µm x 25 cm C18 2 µm 100 Å resin; Thermo Fisher Scientific). For data-independent acquisition (DIA), a 120-minute linear gradient was run from 5% to 35% ACN, followed by 5 minutes from 35% to 95% ACN and 10 minutes at 95% ACN, using solvent A (2% ACN, 0.1 % FA) and solvent B (100% ACN, 0.1% FA) at a 300 nl/min flow rate. For DDA, a full MS scan at mass range 400–1600 m/z (resolution 70,000 at 200 m/z) was followed by MS/MS scans (resolution 17,500 at 200 m/z) of the top 15 most intense precursor ions fragmented by higher energy collision induced dissociation (HCD). To trigger MS/MS scan of precursor ions a MS precursor intensity threshold was set to 1.7e4. The automatic gain control was 1e6 with an accumulation time of 100 ms for MS and 60 ms for MS/MS. For DIA, a full MS scan at mass range 400 to 1,200 m/z (resolution 70,000 at 200

m/z) was followed by 32 subsequent MS/MS full fragmentation scans (resolution 17,500 at 200 m/z) with an isolation window of 26 m/z and 1 m/z overlap between scans.

Mass spectrometry data analysis

Raw files from DDA and DIA analysis were converted to mzML files using MSconvert. All data analysis and searches were processed through openBIS[5]. X!Tandem was used to search data against the human UniProt FASTA database (version November 2015) with reversed decoy sequences. Fixed modification of cysteine carbamidomethylation and variable modifications of methionine oxidation and proline hydroxylation were included in the searches. Mass tolerance for precursor ions and fragment ions was set to 20 ppm and 50 ppm, respectively. The Generated files were subsequently analyzed using peptideProphet, iProphet and MAYU in the Trans-Proteomic Pipeline (TPP, version 4.7)[6–8]. For quantification of DIA data a spectral library was created based on DDA data through workflows included in openBIS[9], applying spectraST to generate target assays, CLI to calculate 1% FDR for peptides and proteins and TRIC to perform feature alignment[10]. Then, openSWATH was used to analyze DIA data[11]. Using R (version 3.6.1) and R Studio software (version 1.2.1335), prototypic peptides were selected and analyzed using the DEqMS package to identify differentially expressed proteins (adjusted for donor ID and tissue localization) and to generate the volcano plot[12]. Identified proteins were classified depending on their cell surface expression using the Cell Surface Protein Atlas, a database with experimental mass spectrometry-derived evidence of protein surface expression[13]. The mass spectrometry proteomics data have been deposited to the ProteomeXchange Consortium (http://proteomecentral.proteomexchange.org) via the PRIDE partner repository[14] with the dataset identifier PXD029028.

Supplementary References

1. Rolandsson, S. *et al.* Primary mesenchymal stem cells in human transplanted lungs are CD90/CD105 perivascularly located tissue-resident cells. *BMJ Open Respir. Res.* **1**, e000027 (2014).
2. Hallgren, O. *et al.* Altered fibroblast proteoglycan production in COPD. *Respir. Res.* **11**, 55 (2010).
3. Malmström, E. *et al.* Large-scale inference of protein tissue origin in gram-positive sepsis plasma using quantitative targeted proteomics. *Nat. Commun.* **7**, 10261 (2016).
4. Hughes, C. S. *et al.* Ultrasensitive proteome analysis using paramagnetic bead technology. *Mol. Syst. Biol.* **10**, 757 (2014).
5. Bauch, A. *et al.* OpenBIS: A flexible framework for managing and analyzing complex data in biology research. *BMC Bioinformatics* **12**, 468 (2011).
6. Deutsch, E. W. *et al.* A guided tour of the Trans-Proteomic Pipeline. *Proteomics* **10**, 1150–1159 (2010).
7. Deutsch, E. W. *et al.* Trans-Proteomic Pipeline, a standardized data processing pipeline for large-scale reproducible proteomics informatics. *Proteomics - Clinical Applications* **9**, 745–754 (2015).
8. Reiter, L. *et al.* Protein identification false discovery rates for very large proteomics data sets generated by tandem mass spectrometry. *Mol. Cell. Proteomics* **8**, 2405–2417 (2009).
9. Rosenberger, G. *et al.* A repository of assays to quantify 10,000 human proteins by SWATH-MS. *Sci. Data* **1**, 140031 (2014).
10. Röst, H. L. *et al.* TRIC: An automated alignment strategy for reproducible protein quantification in targeted proteomics. *Nat. Methods* **13**, 777–783 (2016).
11. Röst, H. L. *et al.* OpenSWATH enables automated, targeted analysis of data-

independent acquisition MS data. *Nature Biotechnology* **32**, 219–223 (2014).

12. Zhu, Y. DEqMS: a tool to perform statistical analysis of differential protein expression for quantitative proteomics data. (2019). doi:10.18129/B9.bioc.DEqMS

13. Bausch-Fluck, D. *et al.* A mass spectrometric-derived cell surface protein atlas. *PLoS One* **10**, e0121314 (2015).

14. Vizcaíno, J. A. *et al.* The Proteomics Identifications (PRIDE) database and associated tools: Status in 2013. *Nucleic Acids Res.* **41**, (2013).

Supplementary Figure S1. Cell-surface marker candidate discovery by exploring the proteome of cultured mesenchymal cells. (**A**) Schematic of the experimental design to identify cell-surface marker candidates. Mesenchymal cells isolated from both airways and lung parenchymal tissues were cultured using two different protocols and analyzed with mass spectrometry to identify cell-surface marker candidates. Cell-surface expression of identified proteins was validated by flow cytometry analysis on culture expanded mesenchymal cells from lung parenchyma. (**B**) Volcano plot of proteomic mass spectrometry data displaying differentially expressed proteins between primary cultured mesenchymal cells isolated with protocol 1 (airways n=4, parenchyma n=4; passage 4) and protocol 2 (airways n=4, parenchyma n=4; passage 4 and 5). The x-axis represent the protein expression fold change (log2 converted) between protocol 1 and protocol 2, and the y-axis represent the p-value (-log10 converted, adjusted for donor ID and tissue localization) Orange and green dots indicate proteins with a p-value ≤ 0.001. Differentially expressed cell-surface proteins with a p-value ≤ 0.001 are listed with their corresponding gene name and CD number. Orange color represent protein upregulation in protocol 1 and green color represents protein upregulation in protocol 2. (**C**) Flow cytometry analysis of the expression of common mesenchymal markers (CD90, CD105, and CD73), pericyte marker (CSPG4) and cell-surface marker candidates on primary cultured mesenchymal cells (protocol 1: n=3; protocol 2: n=3, n=2 for CD13) from lung parenchyma. Data presented as the difference in geometric mean fluorescence intensity (ΔGMFI) between antibody stained sample and unstained or isotype stained control (bars represents the median). (**D**) Histograms showing the expression of CD10, CD13, and CD26 on primary cultured mesenchymal cells (all passage 5) from one healthy donor as analyzed by flow cytometry. Filled histograms represent unstained controls.

Supplementary Figure S2. Flow cytometry analysis of candidate native mesenchymal markers on primary CD105$^+$CD90$^+$ mesenchymal cells from human lung parenchyma from a patient with chronic lung rejection. (**A**) Multicolor flow cytometry analysis showing gating of CD105$^+$CD90$^+$ mesenchymal cells in freshly isolated lung cells from one patient with chronic lung rejection. The plot is shown after forward-scatter/side-scatter gating, exclusion of dead cells (7AAD), hematopoietic cells (CD45) and endothelial cells (CD31), and doublet exclusion (area versus height in forward-scatter). (**B**) Plots show candidate marker expression (x-axis) versus side-scatter (y-axis) in CD90$^+$CD105$^+$ mesenchymal cells. For markers CD10, CD13, and CD26, gates are set to define separated population based on marker expression profile. For markers CD44, CD166 and CSPG4, gates are set based on fluorescence minus one (FMO) controls. Percentage of cells in each gate out of all cells in the plot, is indicated next to the gates. Abbreviations: SSC-A = side scatter-area.

Supplementary Figure S3. CFU-f frequency of index sorted CD105$^+$CD90$^+$CD13$^-$ and CD105$^+$CD90$^+$CD13$^+$ mesenchymal cells in 96-well plates (1 cell/well). CFU-f frequency data (healthy donor n=4) is presented as mean (with SD).

Supplementary Figure S4. (**A**) Top 5 enriched terms for biological processes and molecular functions, respectively, from gene ontology (GO) enrichment analysis of the 191 signature genes. (**B**) Expression of 47 matrisome genes among the signatures genes in CD13$^-$ and CD13$^+$ cells, ordered after matrisome group. Abbreviations: BP: biological process, MF: molecular function.

Supplementary Figure S5. Conformity of cluster annotation with markers used in other publications. (**A**, **B**) Dot plots showing gene expression in clusters from re-analyzed single cell

RNA-seq data of the markers used to annotate mesenchymal cell populations in the original publication by Tsukui et al. (**A**), and in the publication by Travaglini et al. (**B**). Grey boxes indicate the cluster annotation used in each publication. Dot size represent the percentage of expressing cells in each cluster and dot color represent the scaled average expression of each cluster.

Supplementary Figure S6. (**A**) Number of DEG between single cell clusters that are enriched in $CD13^-$ and $CD13^+$ gene signatures, respectively. (**B** and **C**) Dot plots showing the expression of all overlapping signature genes in single cell clusters divided into $CD13^+$ enriched (**B**) and $CD13^-$ enriched (**C**). (**D**) Dot plot showing the expression of *IL33* in mesenchymal clusters. Dot size represent the percentage of expressing cells in each cluster and dot color represent the scaled average expression of each cluster.

Supplementary Figure S7. Re-analysis of single cell RNA-seq dataset of all lung cells ($CD235a^-$ cells) from Tsukui *et al.* (GSE132771). (**A**) UMAP plot showing unsupervised clustering of CD235a- cells from 3 normal lungs. Major cell clusters identified are annotated by color. Mesenchymal cell clusters (pericytes/smooth muscle cells and fibroblasts) are encircled by the dashed line. (**B**) UMAP plots showing the expression of lineage markers used to identified lung cell types. Markers: EPCAM (pan-epithelial), AGER (alveolar type 1 cells), SFTPC (alveolar type 2 cells), PECAM1 (pan-endothelial), PTPRC (pan-hematopoietic), CD3E (T cells), S100A8 (monocytes), CD68 (macrophages), CD83 (dendritic cells).

Supplementary Figure S8. Identification of *MFAP5*$^+$ cells in human lung tissue. (**A**-**K**) Images of sections from distal tissue from normal human lung probed by RNA *in situ* hybridization for *MFAP5*. *MFAP5*$^+$ cells in tunica adventitia of blood vessels (**A**-**G,** images from 3 healthy

donors), interlobular septa (**H** and **I,** images from 2 healthy donors with identifiable interlobular septa) and visceral pleura (**J** and **K**, images from 2 healthy donors). Dashed lines indicate border between tunica adventitia and tunica media in **A-G** and mark interlobular septum in **H** and **I**. The scale bar is 100 µm. Black rectangle inserts are magnifications of areas inside blue rectangles. Abbreviations: lu = lumen, p = pleura.

Supplementary Figure S9. (**A-D**) Images alveolar tissue from sections of normal human lungs probed by RNA *in situ* hybridization for *MFAP5*. *MFAP5*$^+$ cells were not present in alveolar tissue (**A-D**, images from 4 healthy donors). The scale bar is 100 µm.

Supplementary Tables and Figures

Supplementary Table S1. Donor and patient tissue information

Donor/patient no.	Reason for lung explant	Lung disease	Smoking history	Sex	Age at explant (years)
1	Lung transplant (donor)	No	Yes	Male	43
2	Lung transplant (donor)	No	Yes	Male	37
3	Lung transplant (donor)	No	Yes	Male	66
4	Lung transplant (donor)	No	No	Male	68
5	Lung transplant (donor)	No	Yes	Male	26
6	Lung transplant (donor)	No	Yes	Male	66
7	Tumor resection	Non-small-cell lung carcinoma	Yes	Female	64
8	Lung transplant (recipient)	Chronic lung allograft dysfunction	-	Female	54

Supplementary Table S2. Antibodies used for flow cytometry and FACS.

Antibody/dye	Clone	Company	Catalog no.
CD10-BV421	HI10a	BD Biosciences	562902
CD105-APC	266	BD Biosciences	562408
CD105-BV421	266	BD Biosciences	563920/566265
CD105-PE	266	BD Biosciences	560839
CD13-PE	WM15	BD Biosciences	560998/555394
CD166-BV421	3A6	BD Biosciences	562936
CD235a-PE-Cy5	GA-R2	BD Biosciences	559944/561776
CD26-APC	M-A261	BD Biosciences	563670
CD31-FITC	WM59	BD Biosciences	555445/560984
CD44-APC	G44-26	BD Biosciences	560890
CD45-FITC	HI30	BD Biosciences	555482
CD45-PE-Cy7	HI30	BD Biosciences	560915
CD73-PE	AD2	BD Biosciences	550257
CD90-APC	5E10	BD Biosciences	561971
CD90-PE	5E10	BD Biosciences	555596
CD90-PerCP-Cy5.5	5E10	BD Biosciences	561557
CSPG4-PE	9.2.27	BD Biosciences	562415
EpCAM-PerCP-Cy5.5	EBA-1	BD Biosciences	347199

Supplementary Table S3. Signature genes for CD13- and CD13+ populations.

CD13- signature (Padj < 0.001 & log2FC < -1)			CD13+ signature (Padj < 0.001 & log2FC > 1)		
gene	log2FoldChange	padj	gene	log2FoldChange	padj
SCN7A	-1.48	5.74E-29	SEMA3C	1.79	1.13E-30
A2M	-1.52	4.34E-22	COL12A1	1.40	6.16E-26
MALAT1	-1.20	8.79E-22	PCSK6	3.20	6.52E-21
DTNA	-2.50	2.18E-17	ANPEP	2.68	6.52E-21
CA3	-3.48	1.86E-16	AADACL2	3.01	1.23E-17
GPC3	-1.72	3.58E-15	C17orf58	2.28	2.18E-17
TCF21	-1.38	6.64E-15	AIF1L	2.61	1.86E-16
ACTA2	-1.91	2.94E-14	MFAP5	1.48	3.99E-16
FXYD6	-1.44	1.79E-13	HTRA3	1.83	5.32E-15
NPNT	-2.46	3.95E-13	PTGS1	2.05	6.26E-15
EDNRB	-1.87	7.70E-12	PROCR	1.41	1.16E-14
COL15A1	-1.14	7.06E-11	CXCL1	1.44	1.16E-14
CNN1	-1.50	1.57E-10	LVRN	2.60	3.36E-14
H4C3	-1.36	2.13E-10	ALDH1A3	1.74	1.72E-12
CDH11	-1.01	2.19E-10	CLEC3B	1.44	2.10E-12
CDC42EP3	-2.23	2.69E-10	TRAC	1.27	8.07E-12
MYH10	-1.02	1.41E-09	ACKR3	1.42	9.69E-12
MYLK	-1.82	2.11E-09	SLPI	1.70	1.20E-11
TBX2	-1.70	2.41E-09	CREB5	1.14	1.55E-10
TPM2	-1.25	2.44E-09	PRG4	2.00	1.55E-10
DES	-5.13	9.60E-09	SFRP4	1.13	1.55E-10
CCL19	-2.35	9.76E-09	PTHLH	2.32	2.13E-10
PLVAP	-6.17	2.38E-08	PXDNL	5.08	2.13E-10
TM4SF1	-2.37	3.04E-08	LGR4	1.33	4.00E-10
TRIB2	-1.19	4.70E-08	CXCL14	1.68	7.06E-10
PARM1	-1.52	6.11E-08	PCOLCE2	1.08	8.31E-10
ITPR1	-1.48	6.59E-08	STXBP6	1.77	1.41E-09
PRKG1	-1.23	7.15E-08	GFPT2	1.13	1.41E-09
MOXD1	-1.01	9.62E-08	CPXM2	1.58	3.29E-09
ADAMTS8	-3.32	1.08E-07	GPR1	2.97	4.33E-09
CDH13	-1.87	1.45E-07	EMILIN2	1.90	6.93E-09
RAMP1	-2.27	1.74E-07	C1QTNF3	1.28	1.36E-08
IGKC	-2.58	1.92E-07	UAP1	1.05	1.48E-08
MMRN1	-6.91	3.50E-07	CD55	1.07	1.69E-08
CCBE1	-2.09	5.22E-07	PI16	1.23	1.96E-08
DKK3	-1.16	6.04E-07	HUNK	5.82	2.18E-08
SLC38A5	-1.21	8.87E-07	LINC01028	1.50	2.63E-08
MAL	-2.09	1.04E-06	DPP4	2.32	3.24E-08
FBLIM1	-1.29	1.34E-06	ADAMTS5	1.63	3.58E-08
CD82	-1.04	1.65E-06	RAMP2	1.40	3.80E-08
ANKRD36	-1.06	2.10E-06	MATN4	6.21	6.06E-08
FGFR4	-2.68	2.13E-06	PLA2G2A	1.16	6.78E-08
MYH11	-3.55	2.28E-06	NTN4	1.64	1.06E-07
RGS3	-1.49	2.38E-06	ITM2A	1.09	1.45E-07
TAGLN	-1.18	2.89E-06	ADAMTSL1	1.65	2.64E-07
COL6A5	-6.00	3.69E-06	FBN1	1.20	4.88E-07
S1PR1	-2.39	4.30E-06	MAP1LC3C	1.77	7.36E-07
SYNGR2	-1.43	5.15E-06	TNNT3	1.96	1.20E-06
CSRP1	-1.00	7.70E-06	EFHD1	1.63	2.08E-06
ANGPT1	-1.33	1.23E-05	HSD3B7	1.05	2.20E-06
ABCB1	-4.05	1.24E-05	PCSK5	1.25	2.28E-06
SORT1	-2.06	1.66E-05	VIT	1.33	2.35E-06
TNFRSF19	-1.08	1.66E-05	GREB1L	3.57	3.41E-06
CASS4	-2.86	1.78E-05	CNTN4	1.26	5.46E-06
ITGA1	-1.36	1.83E-05	FABP3	1.19	6.27E-06
INPP4B	-2.79	2.18E-05	CD34	1.10	8.81E-06
CARMN	-2.75	2.56E-05	LSP1	1.38	9.74E-06
PLCB4	-3.43	3.30E-05	ADGRD1	1.08	1.13E-05
COL21A1	-1.92	3.53E-05	SIX2	1.73	1.23E-05
LDLRAD3	-1.08	3.55E-05	LINC01133	1.79	1.29E-05
EDNRA	-2.48	4.17E-05	PLEKHG1	2.41	1.31E-05
H1-4	-1.60	4.60E-05	BDKRB2	1.42	1.31E-05
ANKRD36B	-1.12	4.60E-05	HRH1	1.38	1.46E-05
ITGA2	-2.48	5.00E-05	GALNT16	1.38	1.69E-05
ETV1	-1.36	6.84E-05	DIPK1A	1.02	1.78E-05
SNAI1	-1.10	7.22E-05	LRRC8C	1.33	1.83E-05
ADAMTS12	-1.98	7.90E-05	WNT10B	2.48	2.85E-05
NOTCH3	-1.16	8.80E-05	BASP1	1.05	3.01E-05
SPINT2	-2.33	8.99E-05	SPRYD3	1.24	3.77E-05
DYSF	-6.28	0.00011186	GALNT10	1.17	3.78E-05
HSPA7	-1.03	0.00012029	CRLF1	1.05	4.15E-05
ABLIM3	-1.06	0.00013049	SFRP2	1.19	4.60E-05
STON1	-1.59	0.00013449	CD24	3.55	6.92E-05
MRVI1	-2.70	0.00015907	SCARA5	1.03	7.93E-05
MCTP2	-2.07	0.00021789	FBLN2	1.14	8.57E-05
AC130466.1	-4.65	0.00049343	CA12	1.32	8.95E-05
FLI1	-2.47	0.00052037	NTM	1.42	0.00012414
SRGN	-1.16	0.00052037	TLR5	1.15	0.00012551
PPP1R14A	-2.32	0.00056329	APCDD1	1.01	0.00013449
DGKH	-1.83	0.00056603	PXN	1.02	0.00013449
SEPTIN4	-1.30	0.00058394	BMP7	2.45	0.00018146
IGFN1	-2.89	0.00064111	PDE10A	1.76	0.00018648
LAMA5	-1.45	0.00065437	DACT2	10.07	0.00020185
FRMD4B	-1.20	0.00072324	DDIT4L	6.86	0.00022786
ENPP2	-1.63	0.00076296	APBB1IP	1.07	0.00022786
PIEZO2	-1.19	0.00078097	ISM1	1.09	0.0002751
CNR1	-3.04	0.00084018	TMTC1	1.28	0.00030561
ART4	-2.55	0.0008433	GALNT12	1.27	0.00033939
ADAMTS9	-2.13	0.00092779	TNFSF9	1.21	0.00037101
NPR3	-2.51	0.00093573	CALCRL	1.40	0.00037101
EDIL3	-1.66	0.00095012	SDK1	1.12	0.00052045
ZNF66	-2.71	0.00098296	TRPV4	4.91	0.00052322
			CDKN2B	1.10	0.00058394
			ZNF221	7.20	0.00064287
			AC023024.1	4.95	0.00076296
			HELLPAR	1.59	0.00078869
			BHMT2	1.11	0.00083785
			CEP152	1.23	0.00087574
			NHSL1	1.88	0.00098296

Supplementary figure S1

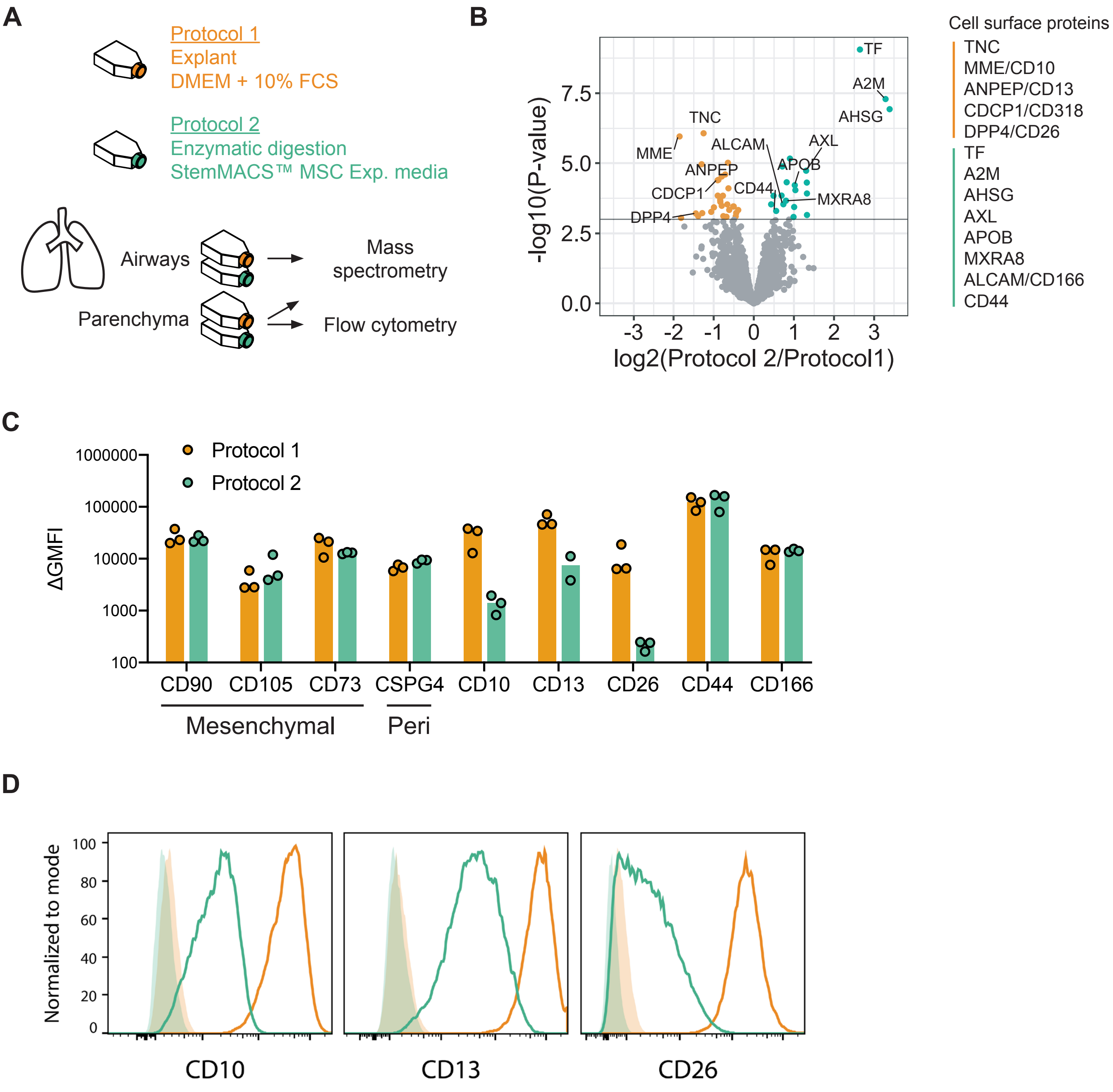

Supplementary figure S2

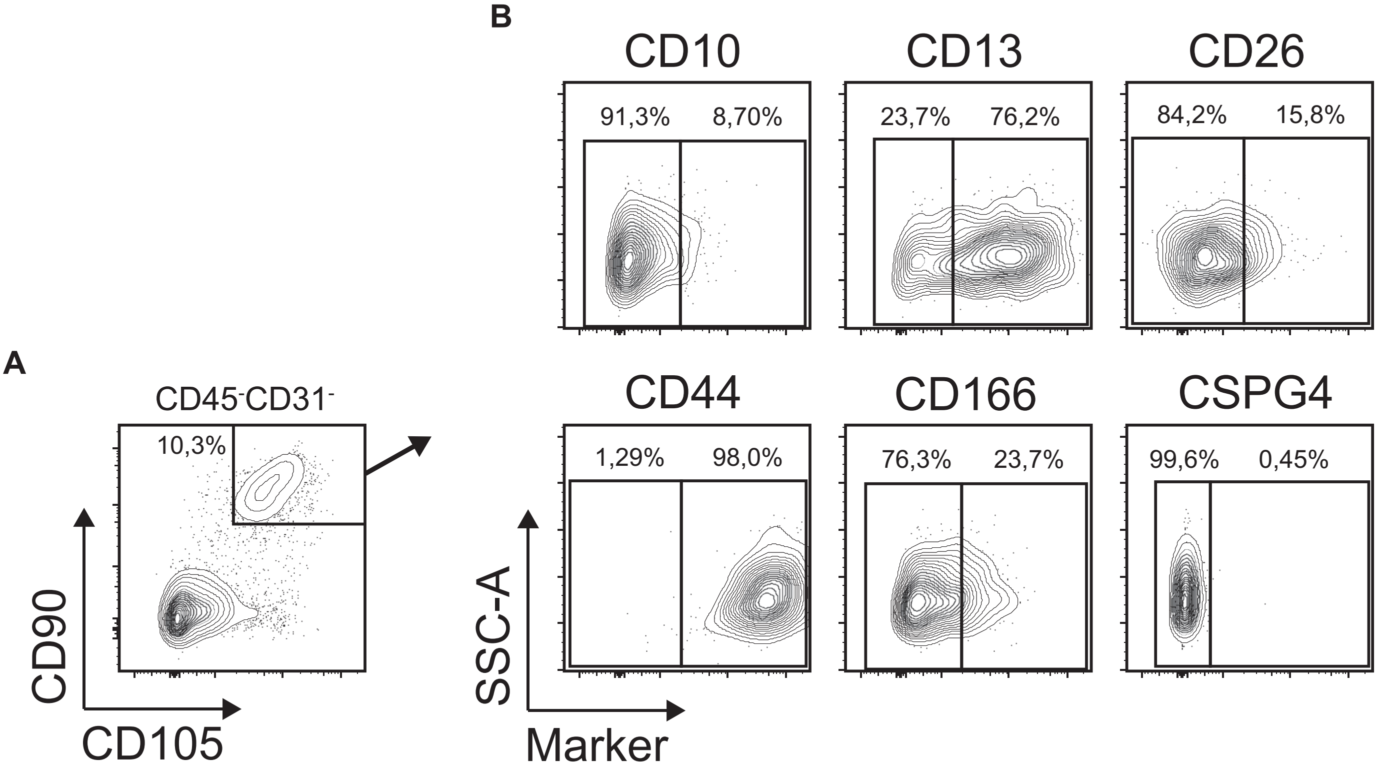

Supplementary figure S3

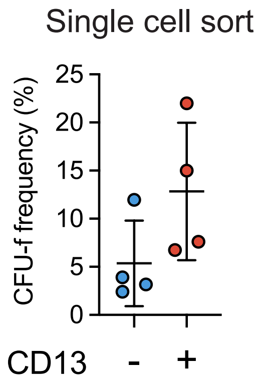

Supplementary figure S4

A

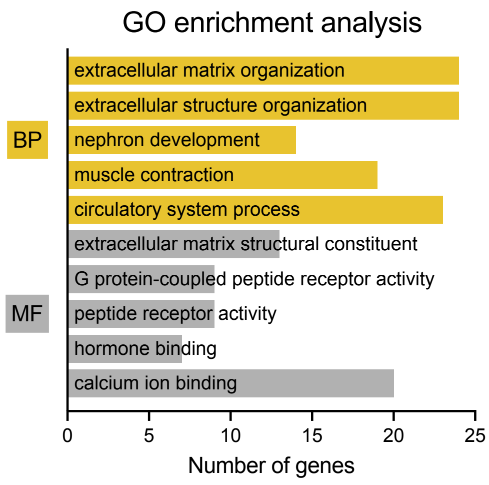

B

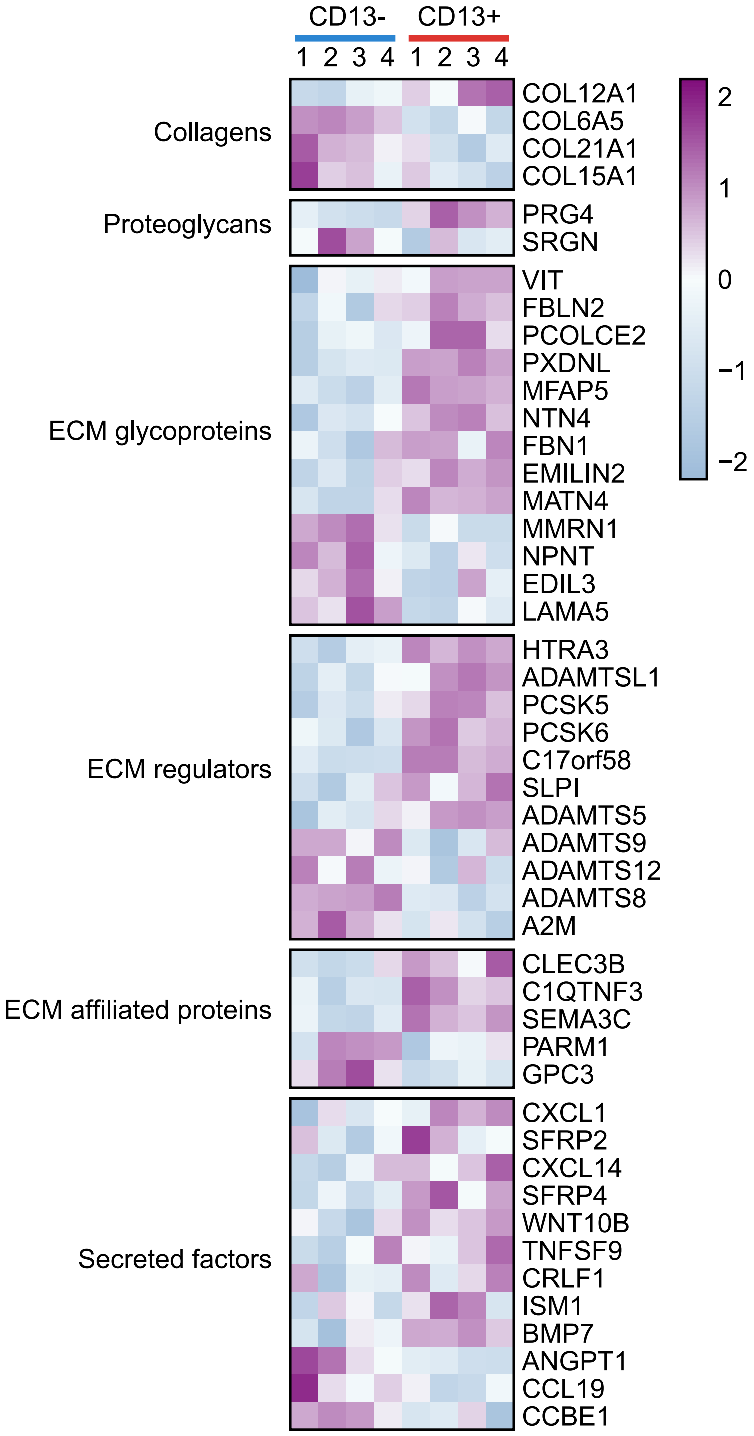

Supplementary figure S5

A Tsukui et al., original cluster annotations

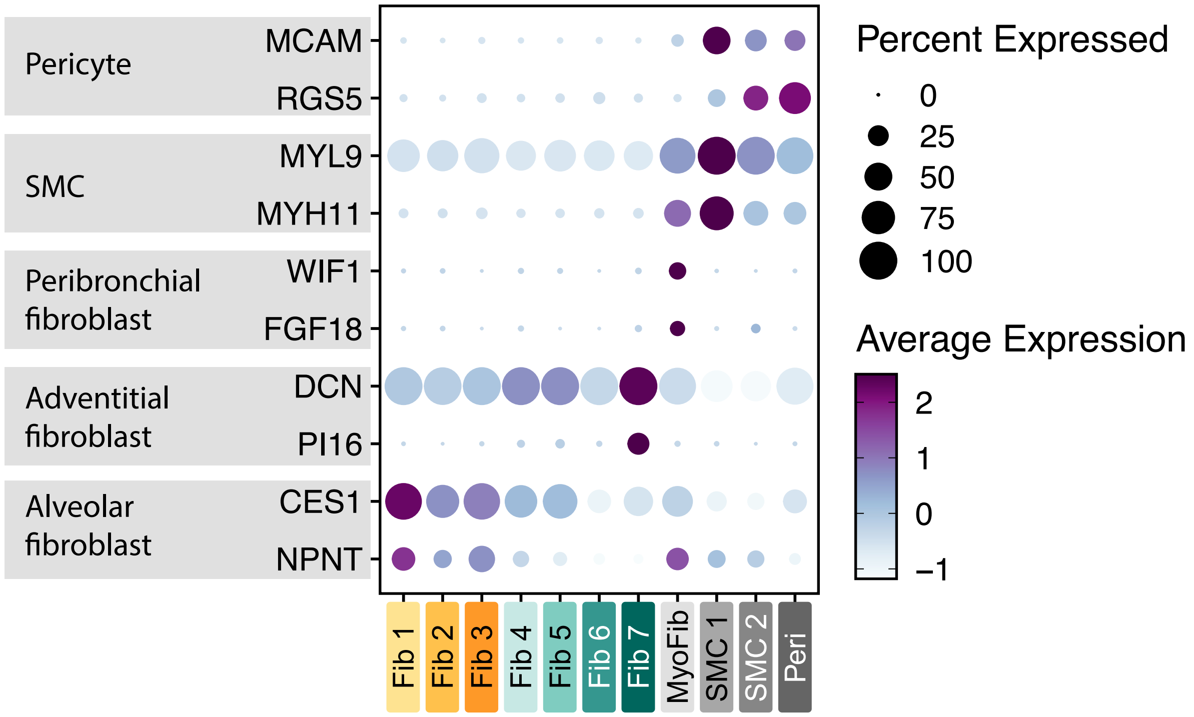

B Travaglini et al., cluster annotations

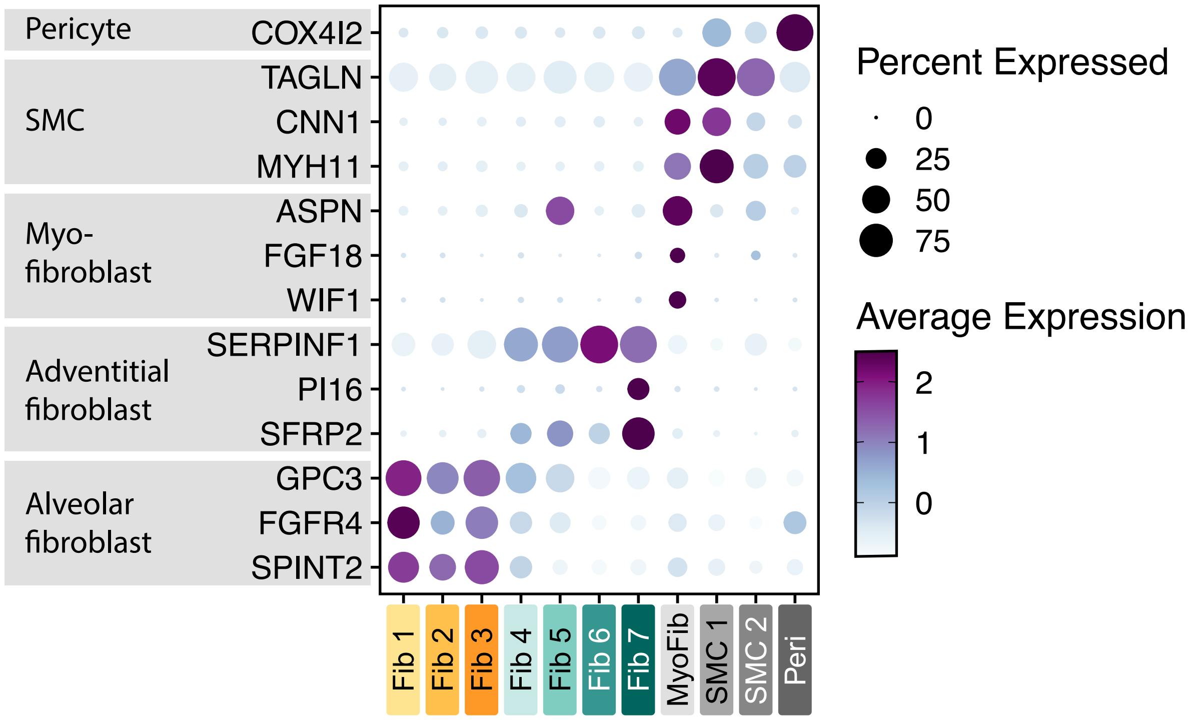

Supplementary figure S6

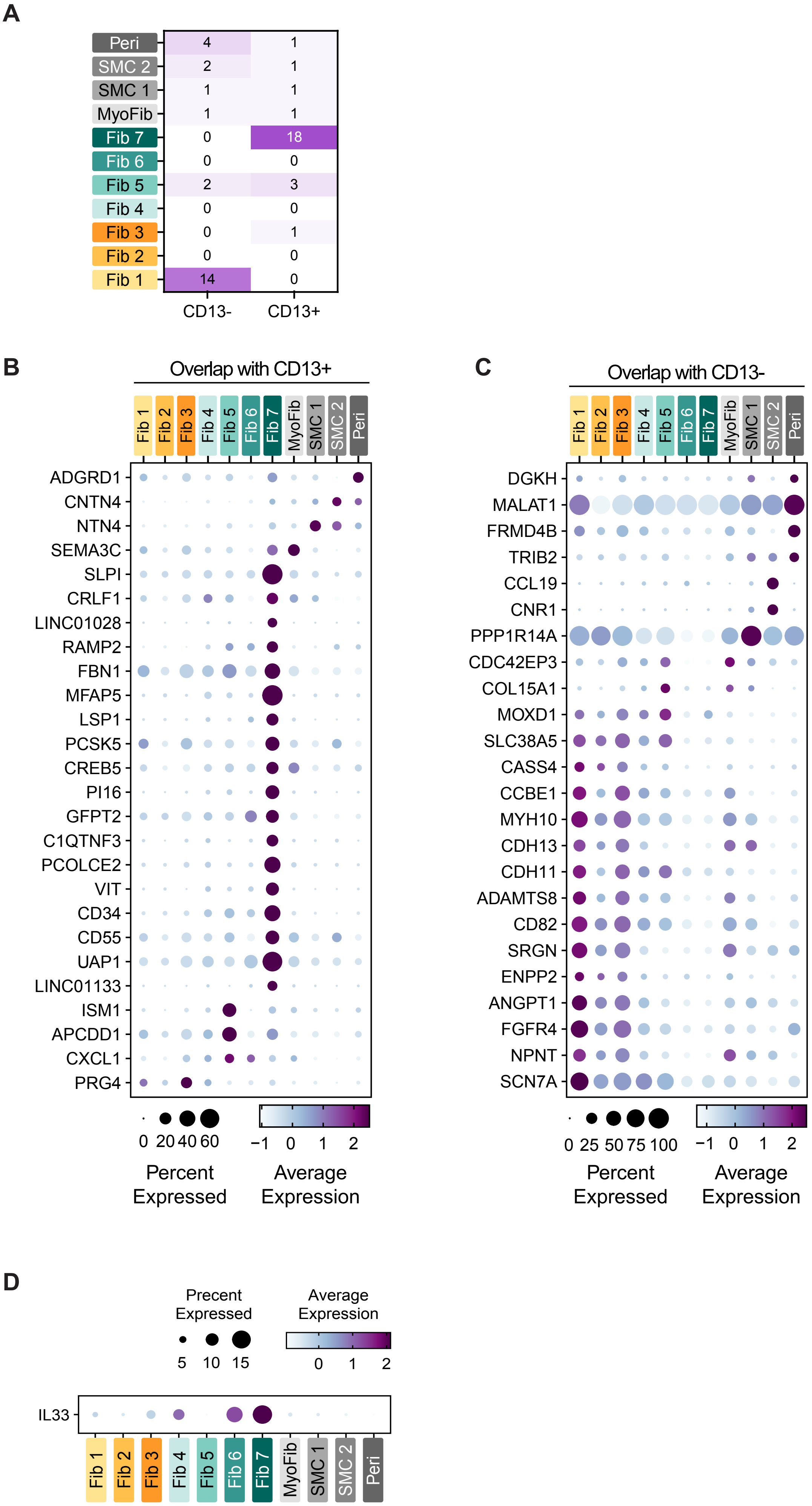

Supplementary figure S7

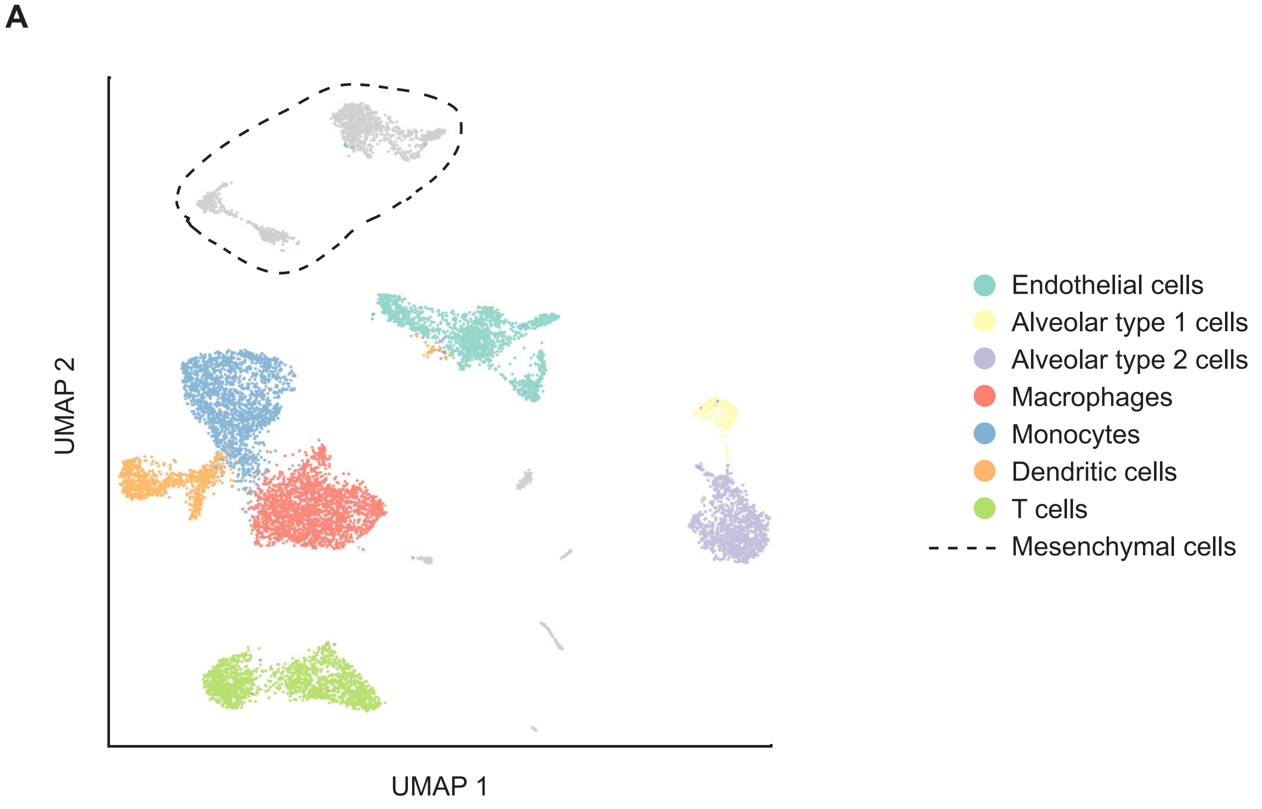

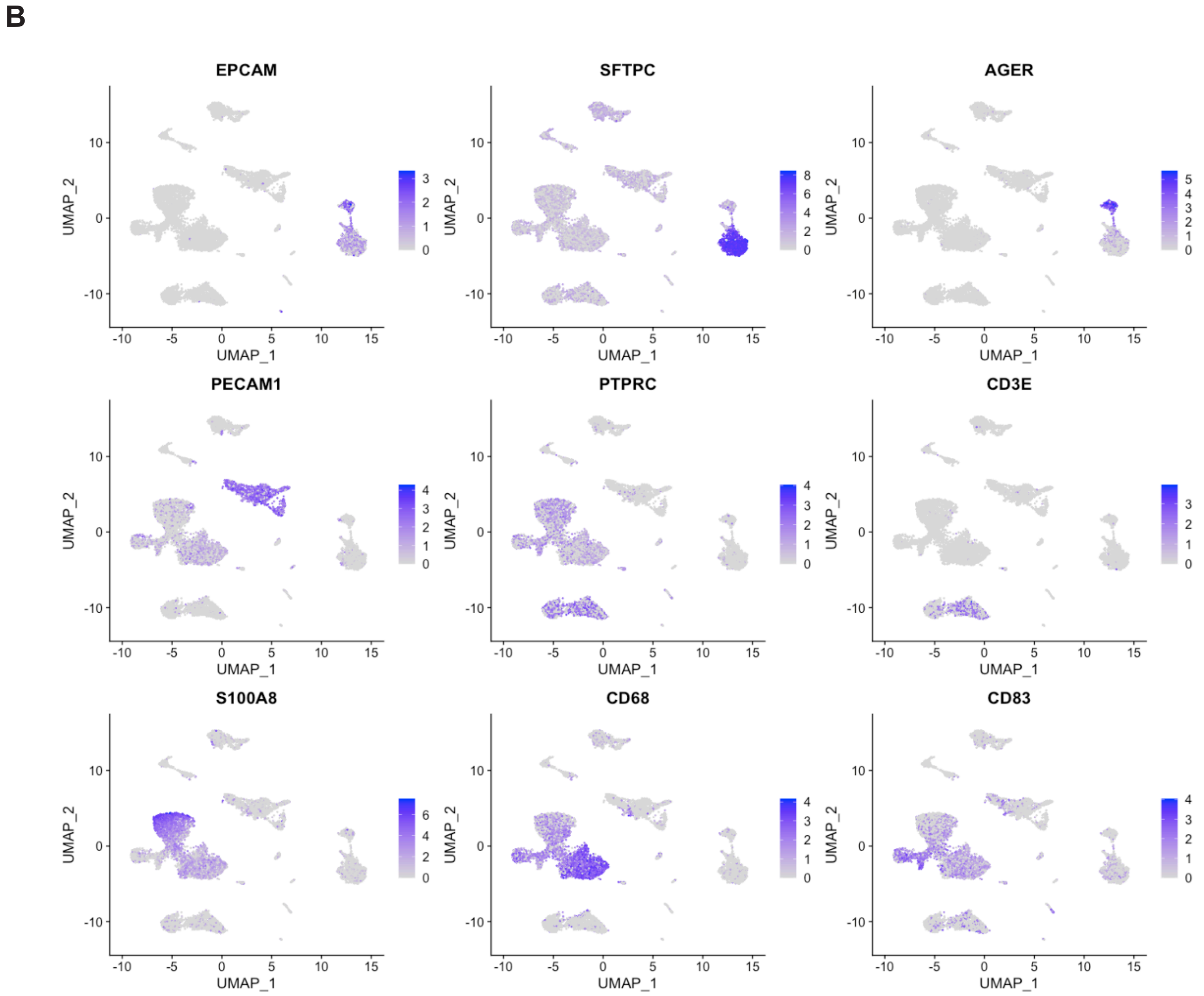

Supplementary figure S8

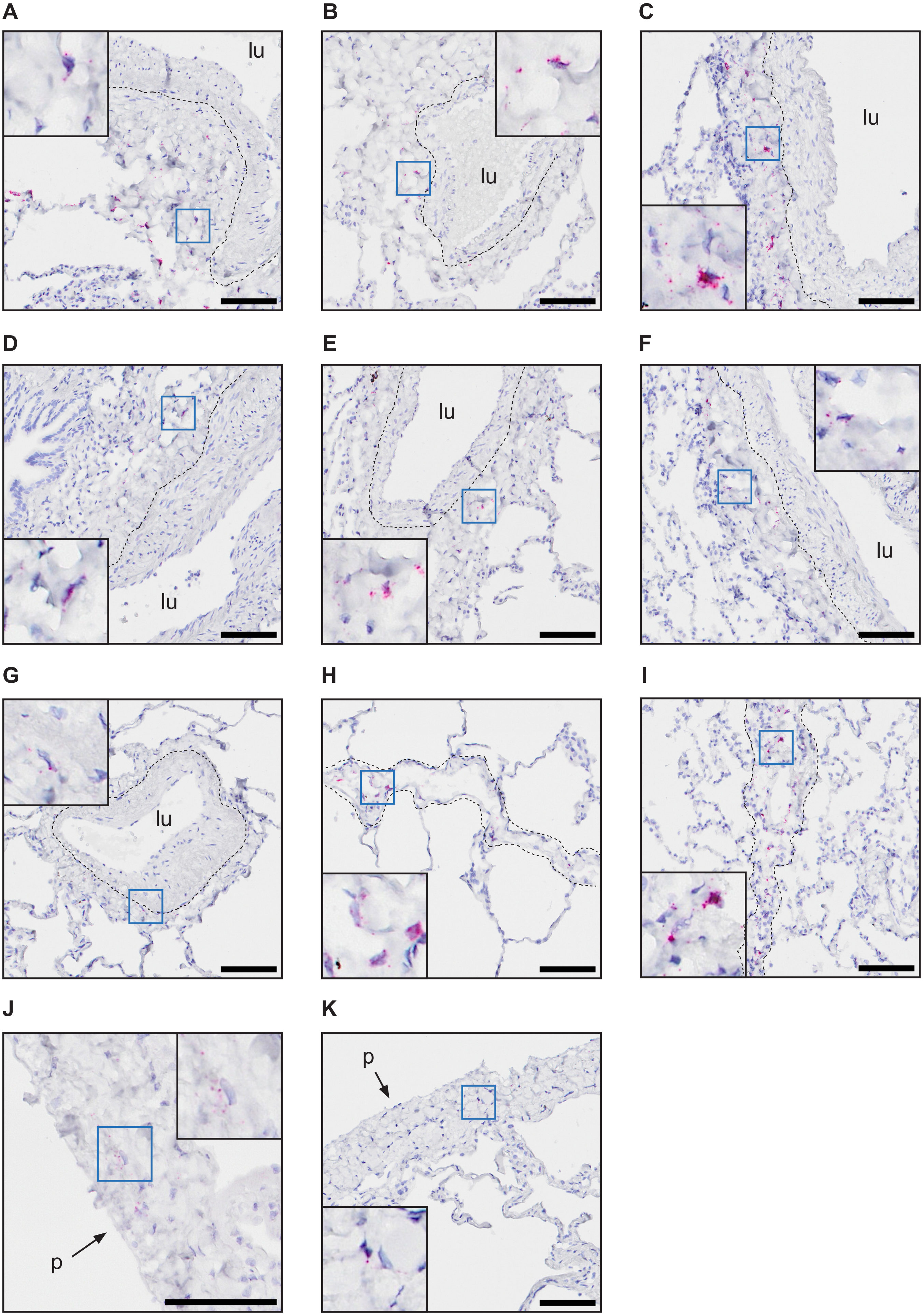

Supplementary figure S9

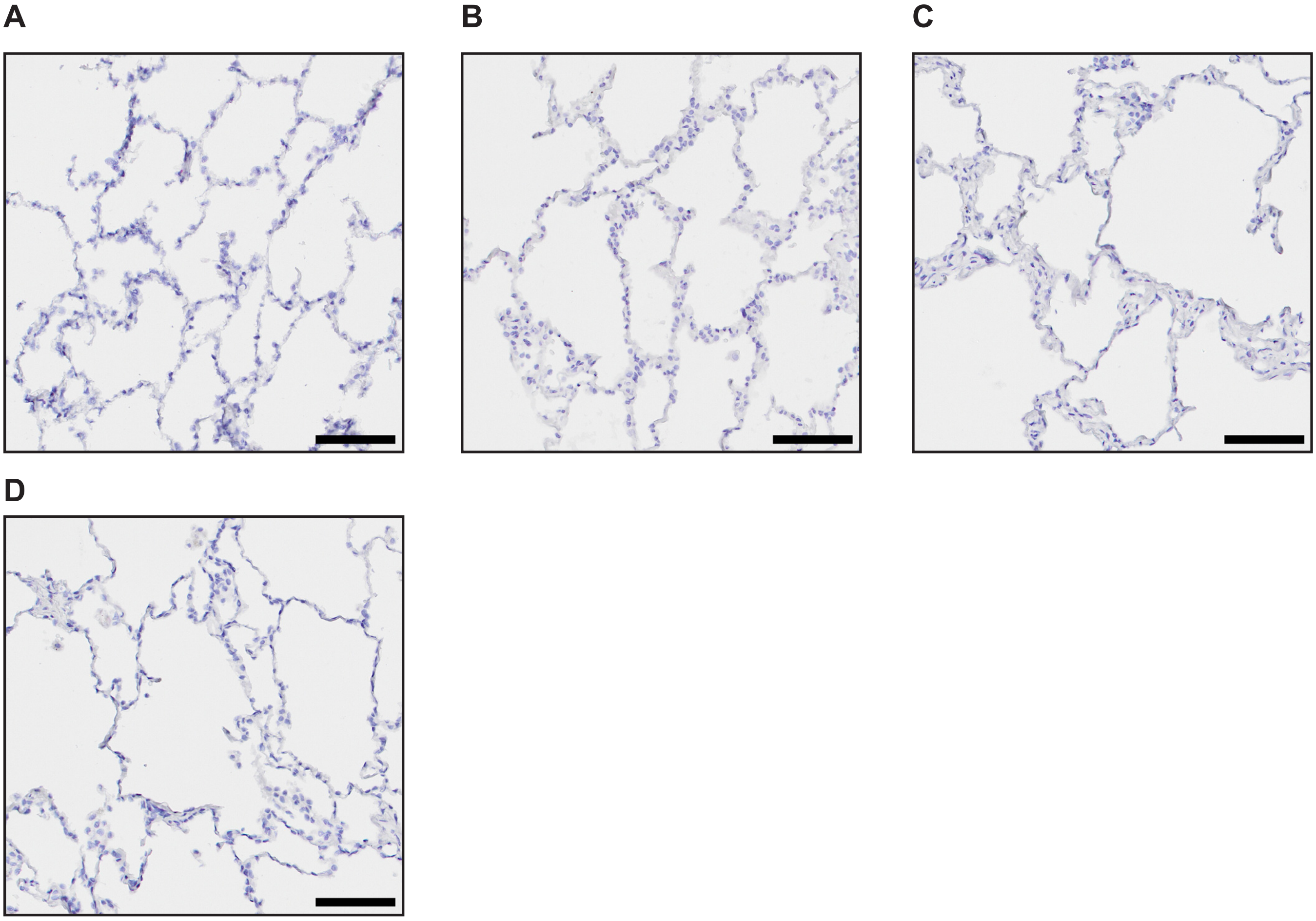

Supplement: Supplementary file 1 — Supplementary Information. [file 41598_2021_3963_MOESM1_ESM.pdf]
